# Phytochemical screening and antibacterial activity of *Skimmia anquetilia* N.P. Taylor and Airy Shaw: A first study from Kashmir Himalaya

**DOI:** 10.3389/fpls.2022.937946

**Published:** 2022-08-12

**Authors:** Masarat Nabi, Nahida Tabassum, Bashir Ahmad Ganai

**Affiliations:** ^1^Department of Environmental Science, University of Kashmir, Srinagar, Jammu and Kashmir, India; ^2^Department of Pharmaceutical Sciences, University of Kashmir, Srinagar, Jammu and Kashmir, India; ^3^Centre of Research for Development, University of Kashmir, Srinagar, Jammu and Kashmir, India

**Keywords:** antibacterial activity, GC-MS, Kashmir Himalaya, multidrug-resistant, plant extracts, *Skimmia anquetilia*

## Abstract

The present study aimed to explore the antibacterial activity of various organic root extracts of *Skimmia anquetilia* N.P. Taylor and Airy Shaw and the identification of major functional groups and phytoconstituents through fourier transform infrared spectrometer (FTIR) and gas chromatography-mass spectrometer (GC-MS). The extracts were evaluated for antibacterial activity against multidrug-resistant (MDR) strains *viz*., *Pseudomonas aeruginosa* (MTCC424), *Escherichia coli* (MTCC739), *Klebsiella pneumoniae* (MTCC139), *Salmonella typhi* (MTCC3224), and *Staphylococcus aureus* (MTCC96). ESKAPE pathogens such as *S. aureus*, *K. pneumoniae*, and *P. aeruginosa* are responsible for a majority of all healthcare acquired infections. The ethyl acetate extract showed the highest zone of inhibition against *P. aeruginosa* (18 mm) followed by *S. aureus* (17 mm). The minimum inhibitory concentration (MIC) of ethyl acetate extract against strain of *S. aureus* (4 mg mL^–1^) demonstrated therapeutically significant antibacterial activity. The FTIR spectra of root extracts revealed the occurrence of functional characteristic peaks of alcohols, carboxylic acids, aromatic compounds, alkanes, alkenes, and amines that indicates the presence of various metabolites in the extracts. The GC-MS investigation led to the identification of diverse phytoconstituents in each of the extracts with varying concentrations and molecular masses. The highest number of compounds were identified from the methanol extract (112), followed by *n*-hexane extract (88) and ethyl acetate extract (74). The most predominant compounds were 5, 10-pentadecadien-1-ol, (*Z,Z*)-(33.94%), *n*-hexadecanoic acid (13.41%) in *n*-hexane extract, 5,10-pentadecadien-1-ol, (*Z,Z*)-(10.48%), 1-hexyl-2-nitrocyclohexane (7.94%) in ethyl acetate extract, and 1-hexyl-2-nitrocyclohexane (15.43%), *cis,cis,cis-*7,10,13-hexadecatrienal (13.29%) in methanol extract. The results of the present study will create a way for the invention of plant-based medicines for various life-threatening microbial infections using *S. anquetilia*, which may lead to the development of novel drugs against drug-resistant microbial infections.

## Graphical Abstract

Phytochemical analysis and antibacterial activity of *Skimmia anquetilia* root extracts. 
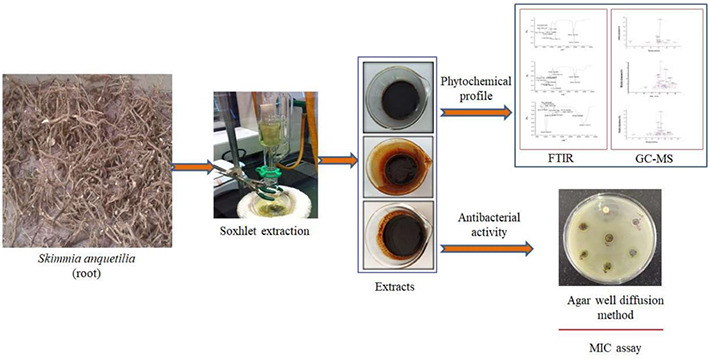

## Introduction

Multidrug-resistant strains of pathogens including *Enterococcus faecium*, *Staphylococcus aureus*, *Klebsiella pneumoniae*, *Acinetobacter baumannii*, *Pseudomonas aeruginosa*, and Enterobacteriaceae (commonly known as ESKAPE pathogens) cause many life-threatening infections. These infections are not treatable with currently available antibiotics if left unaddressed, this will surpass cancer as a cause of death by 2050 (O’Neil, 2014). Even in developed countries like the United States, 23,000 people die each year due to drug-resistant microbial infections. The scenario is similar in Europe and much worse in the developing countries of Asia including India, Latin America, and Africa (Reardon, 2014). Unfortunately, the pipeline for developing novel antibiotics has drained, and clinical approval of new antibiotics is declining. Therefore, it is a need of an hour to discover new lead molecules with a novel mechanism of action and can combat antimicrobial resistance. Further, the use of plant sources and natural products can be used for the rational development of new lead molecules with better efficacy against ESKAPE pathogens. Medicinal plants and natural products have been considered as a great source of medicines to benefit mankind from time immemorial (Benarba and Pandiella, 2020; Javed et al., 2021).

They perform an important role in the prevention of disease and treatment of various ailments across the globe (Banaras et al., 2021). They are a source of many active principles that are both biologically as well as pharmaceutically significant including alkaloids, flavonoids, glycosides, lignans, monoterpenes, lipids (phyto-sterols, toco-pherols, saturated and un-saturated fatty acids), and vitamins (Mazurek et al., 2017; Javaid et al., 2021). Therefore, medicinal plants are a vital source of many drugs, almost a quarter of prescribed medicines (Pan et al., 2013). As per World Health Organization (WHO), nearly three-quarters (80%) of people of developing nations depend upon conventional homeopathic treatments for their basic therapeutic requirements (Ward, 2008). Due to the contribution of various useful phyto-compounds found in various plant components, most medicinal plants are unique in their ability to treat and cure different human diseases (Naqvi et al., 2020). Various medicinal plants (∼80,000 species) have been used as conventional remedies in various indigenous medicine systems in India since ancient times for the treatment of different ailments (Konappa et al., 2020). Currently, almost 25% of active principles have been detected from medicinal plants which are being employed as prescription medicinal products (Konappa et al., 2020; Süntar, 2020). Some studies suggest that around 25,000 of the original plant-specific preparations are available in indigenous folk and conventional medicine systems that are recommended by approximately 15 lakh folk healers for preventive, convincing, and curative purposes (Sen and Chakraborty, 2015). Essential oils and crude extracts of medicinal plants possess numerous kinds of bioactive compounds that have revealed an array of bioefficacies, namely antibacterial (Zeb et al., 2016; Umaru et al., 2019; Nabi et al., 2022a), antifungal (Banaras et al., 2020), antianalgesic (Lisa et al., 2020), antioxidant (Gondwal et al., 2012a; Umaru et al., 2019), anticancerous (Liu Y. T. et al., 2020; Oh et al., 2020), antidiabetic (Tran et al., 2020), etc.

*S. anquetilia* (Rutaceae) is an erect, perennial, glabrous, scented, creeping gregarious, ornamental shrub, 1.5 m in height, found in association with conifers between 1,800 and 2,715 m above msl in Western Himalaya. Traditionally the leaf of the plant has been used in the treatment of headache, smallpox, fever, and also as an anti-inflammatory and antidiabetic agent, etc. *S. anquetilia* is used to treat paralysis, pneumonia, lung cancer, as an insect and pest repellent and as alexipharmic against snake and scorpion poisons (Nabi et al., 2022b). The powdered bark of the plant is used to cure wounds and burn injuries. Apart from its use in conventional medicine, various extracts and bioconstituents of *S. anquetilia* have been broadly used in several treatments such as antioxidants (Prakash et al., 2011; Gondwal et al., 2012a; John et al., 2014), antifeedant (Gondwal et al., 2012b), anti-inflammatory (Kumar et al., 2012), and anticancerous activity (Wani et al., 2016). Although undocumented, the plant is being used to treat diabetes in some areas of Kashmir valley. In recent years, various modern techniques such as FTIR, GC-MS, high-performance liquid chromatography (HPLC), etc., have been widely used to detect functional groups and identify various biologically active curative constituents existing in medicinal plants (Koparde et al., 2019). GC-MS displays molecules extracted at different retention rates with spectral data correlating to secondary metabolites, suggesting fatty acid constituents, whereas, FTIR spectrum indicates absorption peaks with a specific wavelength associated with various functional groups (Sim et al., 2014). To date, no such study has been carried out on the identification of bioactive constituents and antibacterial potential of various root extracts of *S. anquetilia*. Hence, the present study aimed to perform the phytochemical screening for the identification of various functional groups and bioactive constituents through the FTIR and GC-MS techniques and to assess antibacterial activity of *S. anquetilia* root extracts against both the gram-positive and gram-negative bacterial strains.

## Materials and methods

### Chemicals and reagents

All chemicals and reagents used were of analytical grade. *n*-hexane, ethyl acetate, methanol, nutrient agar, mueller hinton broth (MHB), dimethyl sulfoxide (DMSO), and gentamycin were purchased from Merck (Mumbai, India) and Sigma-Aldrich (St. Louis, MO, United States).

### Collection and identification of plant material

*S. anquetilia* used for the investigation was obtained from the Gulmarg area of Baramulla District, Kashmir, India. The plant specimen was authenticated by a leading Taxonomist Dr. Akhter Hussain Malik, Professor, Centre for Biodiversity and Taxonomy (CBT), University of Kashmir. The voucher number is 2697-(KASH).

### Sample extraction

Fresh plant material of *S. anquetilia* was rinsed with running water, dried under shade, and powdered in an electric blender. Dried root powder (50 g) was successively extracted using solvents (each 500 mL) with escalating polarity, namely *n*-hexane, ethyl acetate, and methanol in a Soxhlet extractor. Repetitive extraction of the plant material was carried out before the attainment of colorless solvent. The acquired extracts were then evaporated to dryness using a rotary evaporator and stored at 4°C in airtight glass containers for further analysis.

### Determination of plant extract yield (%)

Yield percentage (w/w) of the dried extracts was calculated as:

Yield (%) = W1 × 100/W2

where W1 is the dry weight of extract after solvent evaporation and W2 is the weight of the dried root powder.

### Fourier transform infrared spectrometer

FTIR spectrometer (Alpha FTIR spectrometer from Bruker optic), fitted with deuterized triglycine sulfate (DTGS) and germanium as a detector and beam splitter, configured to a Windows-based device and coupled to OPUS operating system software (Version 7.0 Bruker optic), was employed throughout the attainment of FTIR spectra. Each sample was placed in direct contact with the attenuated total reflectance (ATR) plate. In spectral regions of 4,000–400 cm^–1^, the FTIR spectra were obtained to determine potential functional groups. The ATR plate was gently wiped with 70% ethanol twice, preceded by drying using soft tissue until filling with the succeeding sample, allowing the ATR plate to dry (Wulandari et al., 2016).

### Gas chromatography-mass spectrometer analysis

GC-MS investigation of *S. anquetilia* root extracts was conducted *via* the Thermo scientific “Chromeleon” (c) Dionex Version: 7.2.8.10783 (Agilent technologies) instrument. GC-MS investigation was performed by employing the following conditions: high electron ionization energy (70 eV) was used. Helium gas (99.99%) was used as the carrier gas with a 1 mL min^–1^ flow rate. Initially furnace temperature was maintained at 50 °C and then increased to 150 °C with a 3 °C min^–1^ increasing rate and retention time of approximately 10 min. The temperature was eventually raised at 10 °C min^–1^ to 300 °C. Then, 1 mL of the sample was kept in a 2 mL screw-top vial in an autoinjector, and 1 μL of the sample was injected in split-mode (1:40). The overall run time of the GC was 33 min. The phytocompounds present in the extracts have been identified based on comparison of their mass spectral patterns with those spectral database of compounds stored in the National Institute of Standards and Technology (NIST) electronic library coupled with the GC-MS system and the data collected has been tabled.

### Antibacterial activity

#### Bacterial strains, media, and controls

Five bacterial strains *viz*., *P. aeruginosa* (MTCC424), *E. coli* (MTCC739), *K. pneumoniae* (MTCC139), *S. typhi* (MTCC3224), and *S. aureus* (MTCC96) were procured from the Microbial Type Culture Collection (MTCC), Chandigarh (India). The strains included both gram-negative as well as gram-positive strains; for agar well diffusion assay, all strains were initially sub-cultured in nutrient agar media and incubated at 37 °C for 18 ± 2 h. The MIC for all strains was determined by the broth dilution method for which they were grown at 37 °C for 18 ± 2 h in MHB. For antibacterial assay, gentamycin (10 μg mL^–1^) and DMSO were used as positive and negative controls whereas for MIC, plant extract and inoculated broth were used as positive and negative controls.

#### Antibacterial screening

Antibacterial efficacy of *n*-hexane, ethyl acetate, and methanol root extracts of *S. anquetilia* was evaluated through the agar well diffusion technique (Clinical and Laboratory Standards Institute, 2008). The nutrient agar media tubes (20 mL) were inoculated with freshly prepared bacterial inoculums using a sterile loop in a back-and-forth motion to ensure an even distribution of inoculums. Petri plates were prepared by pouring pre-inoculated media and allowing it to solidify, and then 8 mm wells were made using a sterile cork borer. A total of 100 μL of different concentrations of each extract and an equal volume of negative control (DMSO) were poured into the wells. The plates were set aside to rest for 30 min to enable the extract to be pre-diffused into the media and were incubated at 37 °C for 17 h. Thereafter, the plates were examined for inhibition zones, and the findings were compared to gentamycin (10 μg mL^–1^).

### Determination of minimum inhibitory concentration

The technique of macro-broth dilution (Clinical and Laboratory Standards Institute, 2008) was used to evaluate the antibacterial potential of ethyl acetate extract by measuring the noticeable bacterial growth in MHB. For MIC estimation in MHB, two-fold serial dilutions of the extract at varying concentrations from 64 to 4 mg mL^–1^ with an optimized concentration of bacterial strains (10^8^ CFU mL^–1^) using 0.5 McFarland standard. The positive control included inoculated broth whereas the negative control included only plant extract and was incubated at 37 °C for 18 h. The MIC is the least concentration of extracts at which the tubes do not show any noticeable growth. To determine the value of MIC, the test tubes were observed for their visible turbidity both pre as well as post-incubation.

### Statistical analysis

All experiments were carried out in three replicates. Data were expressed as mean ± standard deviation and evaluated by analysis of variance (ANOVA). Differences with *p* < 0.05 were considered significant.

## Results

### Physical properties and percent yield

**FIGURE 1:**
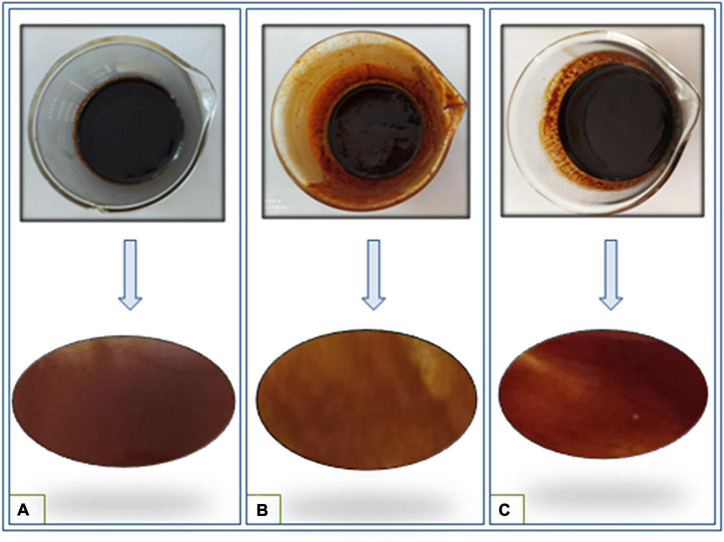
Color variation of different root extracts of *Skimmia anquetilia* **(A)** *n*-hexane extract, **(B)** ethyl acetate extract, and **(C)** methanol extract.

The various extracts possessed varied colors. The extract of *n*-hexane appeared dark brown, the ethyl acetate extract was brown and the methanolic extract was reddish-brown (Figure 1). The methanol extract of *S. anquetilia* had the highest yield (15%), followed by ethyl acetate extract (8.6%), while *n*-hexane extract had the lowest yield (3.3%).

### Fourier transform infrared spectrometer analysis

**FIGURE 2:**
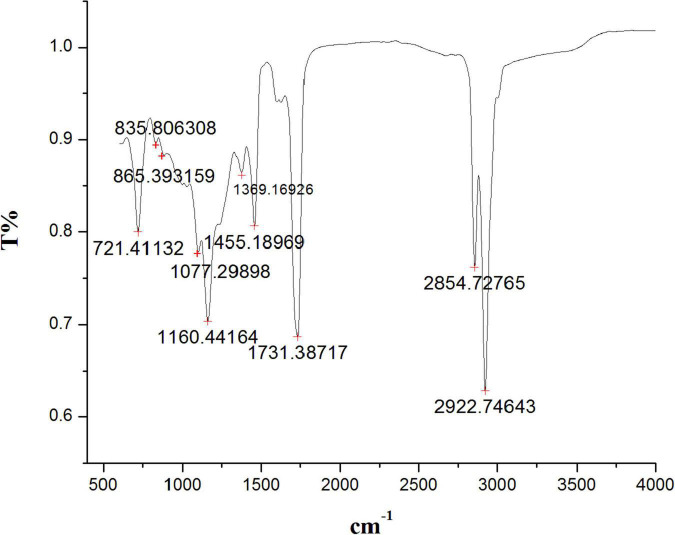
Fourier transform infrared spectrometer (FTIR) spectrum from *n*-hexane root extract of *Skimmia anquetilia*.

The FTIR spectrum indicated the existence of functional groups in the *n*-hexane root extract of *S. anquetilia* with peak positions at 2922.74 cm^–1^, 2854.72 cm^–1^ (alcohols, carboxylic acids, alkanes, and amine salts), 1731.38 cm^–1^ (aromatic compounds, aldehydes), 1455.18 cm^–1^ (aromatics), 1369.16 cm^–1^ (alcohols, phenols), 1160.44 cm^–1^ (amines, tertiary alcohols), 1077.29 cm^–1^ (amines, primary alcohols), 865.39 cm^–1^ (alkenes, aromatics), 835.80 cm^–1^ (alkenes), and 721.41 cm^–1^ (alkenes) (Figure 2 and Table 1).

**TABLE 1:** Fourier transform infrared spectrometer (FTIR) peaks and their assigned functional groups of *n*-hexane root extract of *Skimmia anquetilia*.

S. No.	Wavenumber (cm^–1^)	Compound class	Functional group
1.	2922.74	Alcohol	O-H stretching
		Alkane	C-H stretching
		Amine salt	N-H stretching
		Carboxylic acid	O-H stretching
2.	2854.72	Alcohol	O-H stretching
		Alkane	C-H stretching
		Amine salt	N-H stretching
		Carboxylic acid	O-H stretching
3.	1731.38	Aromatic compound	C-H bending
		Aldehyde	C=O stretching
4.	1455.18	Aromatic compound	C=C stretching
5.	1369.16	Alcohol	O-H bending
		Phenol	O-H bending
6.	1160.44	Amine	C-N stretching
		Tertiary alcohol	C-O stretching
7.	1077.29	Amine	C-N stretching
		Primary alcohol	C-O stretching
8.	865.39	Alkene	C-H bending
9.	835.80	Alkene	C=C bending
10.	721.41	Alkene	C=C bending

**FIGURE 3:**
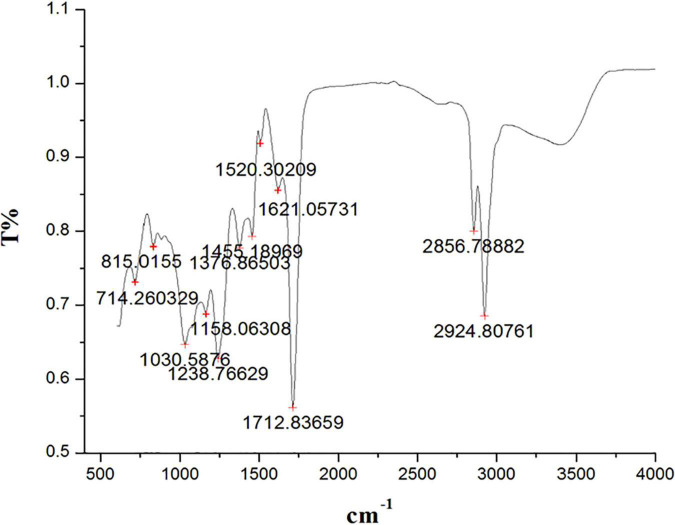
Fourier transform infrared spectrometer (FTIR) spectrum from ethyl acetate root extract of *Skimmia anquetilia*.

The peaks at 2929.80 cm^–1^, 2856.78 cm^–1^ (alcohols, carboxylic acids, alkanes, and amine salts), 1712.83 cm^–1^ (aromatic compounds, aliphatic ketones, and carboxylic acids), 1621.05 cm^–1^ (conjugated alkenes, amines, and cyclic alkenes), 1520.30 cm^–1^ (aromatics), 1455.18 cm^–1^ (aromatics), 1376.36 cm^–1^ (alcohols, phenols), 1238.76 cm^–1^ (amines, alkyl aryl ether), 1158.06 cm^–1^ (amines, tertiary alcohols), 1030.58 cm^–1^ (amines), 815.01 cm^–1^ (alkenes), and 714. 26 cm^–1^ (alkenes) confirmed the presence of functional groups in ethyl acetate root extract (Figure 3 and Table 2).

**TABLE 2:** Fourier transform infrared spectrometer (FTIR) peaks and their assigned functional groups of ethyl acetate root extract of *Skimmia anquetilia*.

S. No.	Wavenumber (cm^–1^)	Compound class	Functional group
1.	2929.80	Alcohol	O-H stretching
		Carboxylic acid	O-H stretching
		Alkane	C-H stretching
		Amine salt	N-H stretching
2.	2856.78	Alcohol	O-H stretching
		Carboxylic acid	O-H stretching
		Alkane	C-H stretching
		Amine salt	N-H stretching
3.	1712.83	Aromatic compound	C-H bending
		Aliphatic ketone	C=O stretching
		Carboxylic acid	C=O stretching
4.	1621.05	Conjugated alkene	C=C stretching
		Amine	N-H bending
		Cyclic alkene	C=C stretching
5.	1520.30	Aromatics	C=C stretching
6.	1455.18	Aromatics	C=C stretching
7.	1376.36	Alcohol	O-H bending
		Phenol	O-H bending
8.	1238.76	Alkyl aryl ether	C-O stretching
		Amine	C-N stretching
9.	1158.06	Amine	C-N stretching
		Tertiary alcohol	C-O stretching
10.	1030.58	Amine	C-N stretching
11.	815.01	Alkene	C=C bending
12.	714. 26	Alkene	C=C bending

**FIGURE 4:**
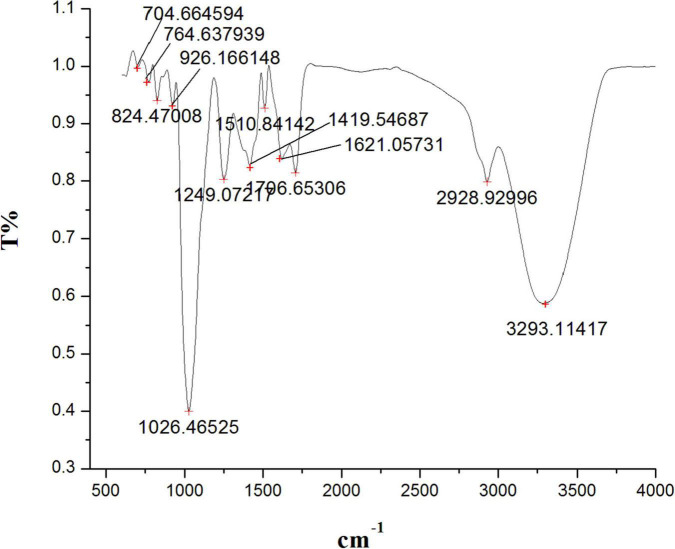
Fourier transform infrared spectrometer (FTIR) spectrum from methanolic root extract of *Skimmia anquetilia*.

Similarly, the FTIR spectra of the methanol root extract of *S. anquetilia* revealed the presence of functional groups with peak ranges at 3293.11 cm^–1^ (alcohols, carboxylic acids, and alkynes), 2928.92 cm^–1^ (alcohols, amine salts, carboxylic acids, and alkanes), 1706.65 cm^–1^ (aromatic compounds, aliphatic ketones, carboxylic acids, conjugated acids, and conjugated aldehydes), 1621.05 cm^–1^ (cyclic alkenes, amines, and conjugated alkenes), 1510.84 cm^–1^ (aromatic compounds), 1419.54 cm^–1^ (carboxylic acids, alcohols, and aromatics), 1249.07 cm^–1^ (acid, alkyl aryl ether, and amines), 1026.46 cm^–1^ (amines, phosphate ion), 926.16 cm^–1^ (alkenes), 824.47 cm^–1^ (alkenes), 764.63 cm^–1^ (-), and 704.66 cm^–1^ (alkenes) (Figure 4 and Table 3).

**TABLE 3:** Fourier transform infrared spectrometer (FTIR) peaks and their assigned functional groups of methanol root extract of *Skimmia anquetilia*.

S. No.	Wavenumber (cm^–1^)	Compound class	Functional group
1.	3293.11	Alcohol	O-H stretching
		Carboxylic acid	O-H stretching
		Alkyne	C-H stretching
2.	2928.92	Alcohol	O-H stretching
		Amine salt	N-H stretching
		Alkane	C-H stretching
		Carboxylic acid	O-H stretching
3.	1706.65	Aromatic compound	C-H bending
		Aliphatic ketone	C=O stretching
		Carboxylic acid	C=O stretching
		Conjugated acid	C=O stretching
		Conjugated aldehyde	C=O stretching
4.	1621.05	Cyclic alkene	C=C stretching
		Amine	N-H bending
		Conjugated alkene	C=C stretching
5.	1510.84	Aromatic compound	C=C stretching
6.	1419.54	Carboxylic acid	O-H bending
		Alcohol	O-H bending
7.	1249.07	Acid	C-O stretching
		Alkyl aryl ether	C-O stretching
		Amine	C-N stretching
8.	1026.46	Phosphate ion	PO_3_ stretching
		Amine	C-N stretching
9.	926.16	Alkene	C=H bending
10.	824.47	Alkene	C=C bending
11.	764.63	Alkene	C=H bending
12.	704.66	Alkene	C=C bending

### Gas chromatography-mass spectrometer analysis

**FIGURE 5:**
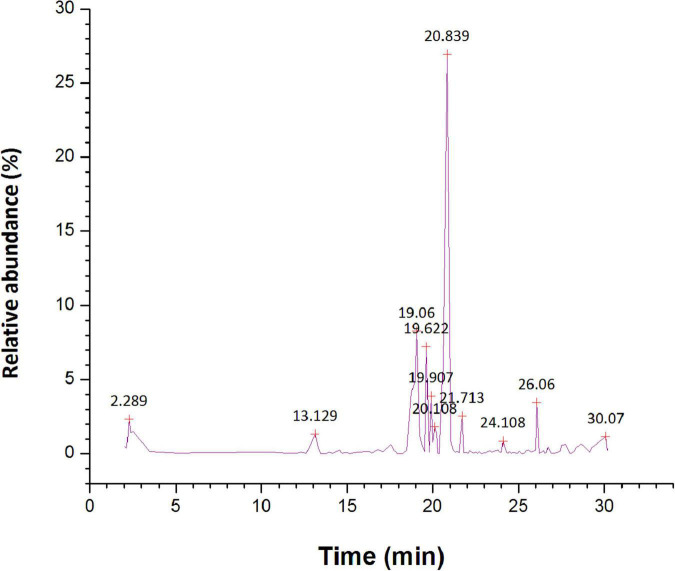
Gas chromatography-mass spectrometer (GC-MS) chromatogram for major compounds of *n*-hexane root extract of *Skimmia anquetilia*.

The GC-MS chromatogram of *n*-hexane, ethyl acetate, and methanol root extracts of *S. anquetilia* recorded a total of 88, 74, and 112 peaks respectively corresponding to the bioactive compounds that were recognized by relating their mass spectral fragmentation patterns to that of the known compounds described by the NIST library. The analysis of *n*-hexane extract *via* GC-MS resulted in the identification of 88 distinct phytoconstituents. The identified chemical constituents according to their retention time, peak area (%), and molecular weight are listed in Supplementary Table 1. The predominant organic constituents that were present in *n*-hexane extract (Figure 5) are 5, 10-pentadecadien-1-ol, (*Z,Z*)-(33.94%), *n*-hexadecanoic acid (13.41%), 8a(2H)-phenanthrenol, 7-ethenyldodecahydro-1,1,4a,7- tetramethyl-, acetate, [4as-(4a.alpha.,4b.beta.,7.beta.,8a.alpha.,10a.beta.)]- (7.30%), 1-hexyl-2-nitrocyclohexane (4.35%), l-alanine, *N*-(3-trifluoromethylbenzoyl)-, heptyl ester (4.02%), cyclopropane, 1-ethyl-2- methyl-, *cis*-(3.91%), squalene (3.59%), 7H-furo(3,2-g)(1)benzopyran-7-one,4,9-dimethoxy- (2.56%), 7H-furo[3,2-g][1]benzopyran-7-one, 4-methoxy-(1.82%), dihydro-*cis*-α-copaene-8-ol (1.78%), and cyclohexane (1.57%).

**FIGURE 6:**
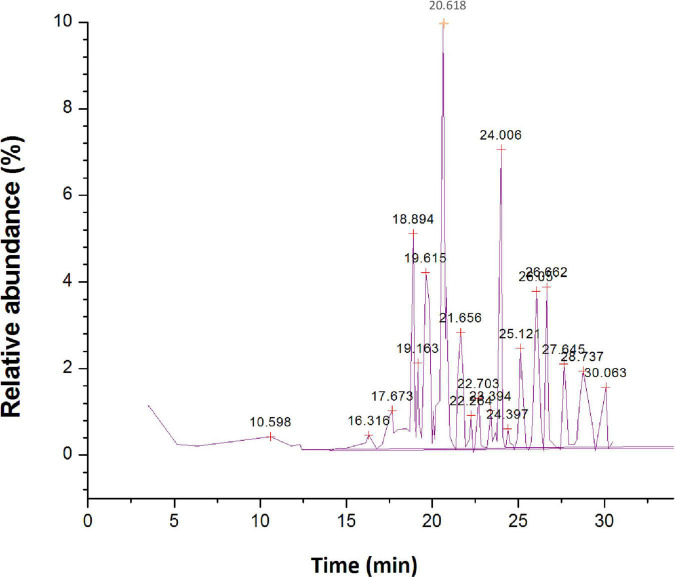
Gas chromatography-mass spectrometer (GC-MS) chromatogram for major compounds of ethyl acetate root extract of *Skimmia anquetilia*.

The GC-MS investigation of ethyl acetate extract led to the detection of 74 different organic constituents (Supplementary Table 2). The most abundant organic compounds found in the extract of ethyl acetate (Figure 6) are 5,10-pentadecadien-1-ol, (Z,Z)-(10.48%), 1-hexyl-2-nitrocyclohexane (7.94%), phthalic acid, di(2-propylpentyl) ester (7.41%), 1-methylene-2b-hydroxymethyl-3,3-dimethyl-4b-(3-methylbut-2-enyl)-cyclohexane (5.59%), *n*-hexadecanoic acid (5.54%), 1,3,3-trimethyl-2-hydroxymethyl-3,3-dimethyl-4-(3-methylbut-2-enyl)-cyclohexene (4.65%), 2-cyclopropen-1-ol, 1,2-dicyclopentyl-3-(1-methylethyl)-, acetate (4.25%), squalene (3.79%), l-alanine, *N*-(3-trifluoromethylbenzoyl)-, isohexyl ester (3.49%), 2R-acetoxymethyl-1,3,3-trimethyl-4t-(3-methyl-2-buten-1-yl)-1t-cyclohexanol (2.87%).

**FIGURE 7:**
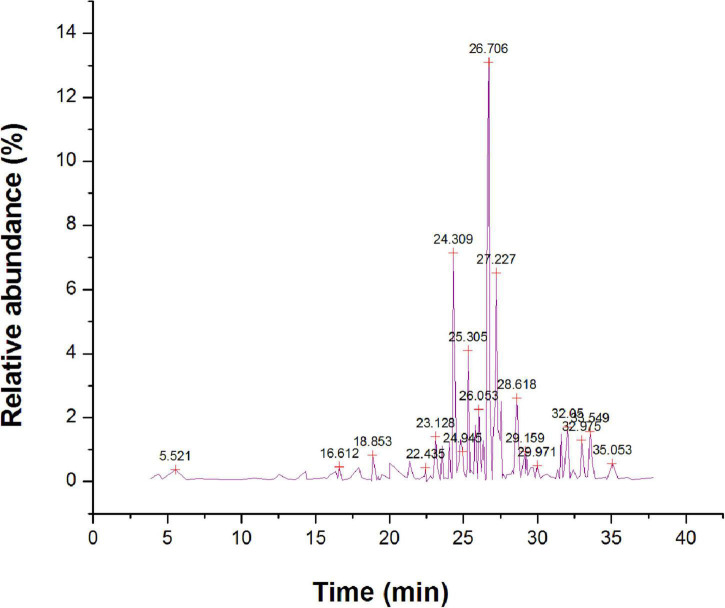
Gas chromatography-mass spectrometer (GC-MS) chromatogram for major compounds of methanol root extract of *Skimmia anquetilia*.

The methanol extract *via* GC-MS investigation had resulted in the detection of 112 different bioactive constituents (Supplementary Table 3). The main organic constituents found in methanol extract (Figure 7) are 1-hexyl-2-nitrocyclohexane (15.43%), *cis*,*cis*,*cis*-7,10,13-hexadecatrienal (13.29%), methyl 9-*cis*, 11-*trans*-octadecadienoate (9.62%), hexadecanoic acid, methyl ester (7.24%), 5, 10-pentadecadien-1-ol, (Z,Z)-(6.28%), tetradecanoic acid, 12 methyl-, methyl ester, (S)-(4.54%), farnesyl butanoate (1.41%), and 7H-furo[3,2-g][1]benzopyran-7-one,4,9-dimethoxy- (2.62%). The bioactive compounds with significant antibacterial activities are presented in Table 4.

**TABLE 4:** Bioactive compounds with significant antimicrobial activity.

S. No.	Compounds	Root extracts			Biological activity	References
		*n*-hexane	Ethyl acetate	Methanol		
1.	1-hexyl-2-nitrocyclohexane	+	+	+	Antimicrobial activity against *Salmonella suis* (ATCC 13076), *Pseudomonas aeruginosa* (ATCC 27583), *Escherichia coli* (ATCC 25922), *Staphyllococcus aureus* (ATCC 25923), *Bacillus subtilis* (ATCC 6633), *Shigella sonnei* (ATCC 11060), and *Candida albicans* (ATCC 10231).	Al-Wathnani et al., 2012
2.	2R-acetoxymethyl-1,3,3-trimethyl-4t-(3-methyl-2-buten-1-yl)-1t-cyclohexanol	+	+	+	Antibacterial activity against *Pseudomonas aeruginosa*, *Escherichia coli*, *Klebsiella pneumoniae*, *Salmonella typhi*, and *Staphylococcus aureus.*	Nabi et al., 2022a
3.	5, 10-pentadecadien-1-ol, (Z,Z)-	+	+	+	Antibacterial activity against *Staphylococcus aureus* and *Pseudomonas aeruginosa.*	Majeed et al., 2021
4.	7H-furo[3,2-g][1]benzopyran-7-one,4,9-dimethoxy-	+	–	+	Antimicrobial activity against *Bacillus subtilis*, *Klebsiella pneumoniae*, *Aspergillus niger*, and *Candida albicans.*	AlMalki, 2016
5.	*n*-hexadecanoic acid	+	+	+	Antibacterial activity against *Bacillus subtilus* (NCIM 2718), *Staphylococcus aureus* (ATCC 25923), *Pseudomonas aurginosa* (ATCC 27853), *Klebseilla pneumoniae* (ATCC 70063), and *Escherichia coli* (ATCC 25922).	Mickymaray et al., 2016
6.	Squalene	+	+	+	Antibacterial activity against *Escherichia coli*, *Klebsiella pneumoniae, Vibrio harveyi Micrococcus roseus*, and *Staphylococcus aureus*.	Dordab et al., 2021
7.	Hexadecanoic acid, methyl ester	–	–	+	Antibacterial activity against *Staphylococcus aureus* (W35), *Pseudomonas aeruginosa* (D31), *Klebsiella pneumoniae* (DF30), *Klebsiella pneumoniae* (B45), and *Bacillus subtilis*.	Lalthanpuii and Lalchhandama, 2019; Shaaban et al., 2021
8.	7-hydroxycoumarin	–	–	+	Antibacterial activity against *Bacillus subtilis*, *Staphylococcus aureus*, *Streptococcus pyogene*, *Pseudomonas aeruginosa*, *Salmonella typhimurium*, *Escherichia coli*, and antifungal activity against strains of *Candida albicans*, *Candida krusei*, *Candida parapsilosis*, and *Cryptococcus neoformans.*	Farshori et al., 2011
9.	Linalool	–	–	+	Antibacterial activity against *Pseudomonas aeruginosa*, and *Shewanella putrefaciens.*	Liu X. et al., 2020; Guo et al., 2021
10.	Geraniol	–	–	+	Antimicrobial activity against *Candida* and *Staphylococcus* genera.	Lira et al., 2020
11.	*cis*,*cis*,*cis*-7,10,13-hexadecatrienal	–	–	+	Antibacterial activity against *Escherichia coli.*	Li et al., 2014
12.	Cyclohexane	+	–	–	Antibacterial activity against *Staphylococcus aureus, Staphylococcus epidermidis, Pseudomonas aeruginosa*, and *Escherichia coli.*	Shoaib et al., 2019
13.	Phthalic acid, di(2-propylpentyl)ester	–	–	+	Antimicrobial activity against bacteria and yeasts, such as *Shigella flexneri*, *Escherichia coli*, *Klebsiella pneumoniae*, *Bacillus cereus*, *Staphylococcus aureus*, *Enterococcus faecalis*, *Bacillus subtilis*, *Candida albicans*, and *Candida glabrata.*	Chakraborty et al., 2022
14.	Methyl 9-*cis*, 11-*trans*-octadecadienoate	–	–	+	Antifungal activity against *Microsporum canis* and *Trichophyton mentagrophytes*.	Ouf et al., 2022
15.	Hexadecanoic acid, ethyl ester	–	+	–	Antibacterial activity against *Aeromonas hydrophila, Edwardsiella tarda*, and *Vibrio ordalli.*	Gohar et al., 2010
16.	Campesterol	+	–	–	Antibacterial activity against the rate limiting enzyme involved in cell wall synthesis of bacteria i.e., glucosamine 6 phosphate synthase (PDB ID – 4VF5) as protein target.	Ranjith, 2019

“+” indicate presence and “–” absence of phytocontituents.

### Antibacterial activity

**FIGURE 8:**
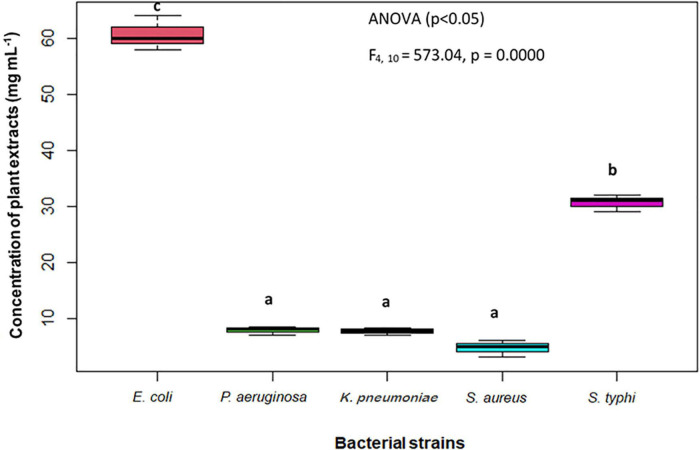
Minimum inhibitory concentration (MIC) of the most effective plant extract against test organisms. Dissimilar letters (b and c) show significant difference and similar letters (a) show insignificant difference.

The findings of the agar well diffusion assay (Table 5) showed that all the extracts are active against the bacterial strains tested. The most effective extract was ethyl acetate extract, which has exhibited the greatest effect against *P. aeruginosa* with an inhibition zone of 18 mm, followed by *S. aureus* and *K. pneumoniae* each having an inhibition zone of 17 mm. The methanol extract displayed a similar trend of bacterial inhibition, with the maximum zone of inhibition against *S. aureus* (17 mm), followed by *K. pneumoniae* (16 mm), and *E. coli* (15 mm). Similarly, the *n*-hexane extract exhibited strong inhibition against *K. Pneumoniae* (17 mm) and *S. typhi* (17 mm) followed by *E. coli* (16 mm). Owing to the increased antibacterial activity in agar well diffusion analysis against the tested bacterial strains, the MIC was evaluated in the ethyl acetate extract of *S. anquetilia*. The ethyl acetate extract showed the MIC of 4 mg mL^–1^ against *S. aureus* (Table 6). Analysis of variance (*P* < 0.05) showed that there is significant difference between the strains with respect to the concentration of plant extracts used. Dissimilar letters show significant difference and similar letters show insignificant difference (Tukey’s HSD test) (Figure 8). The highest antibacterial activity could be attributed to the presence of bioactive constituents in the extracts.

**TABLE 5:** *In-vitro* antibacterial activity of *Skimmia anquetilia* root extracts against tested bacterial strains.

Organic extract	Concentration (mg mL^–1^)	Zone of inhibition (mm) (Mean ± SD)				
		Gram-negative bacteria				Gram-positive bacteria
		*Escherichia coli*	*Pseudomonas aeruginosa*	*Klebsiella pneumoniae*	*Salmonella typhi*	*Staphylococcus aureus*
*n*-hexane	10	14.0 ± 2.64	14.0 ± 2.0	11.0 ± 9.84	14.0 ± 3.0	12.0 ± 1.73
	20	14.0 ± 2.0	13.0 ± 1.0	15.0 ± 3.0	12.0 ± 1.0	13.0 ± 1.0
	40	14.0 ± 3.0	13.0 ± 2.0	17.0 ± 1.0	13.0 ± 2.0	13.0 ± 1.73
	80	16.0 ± 2.64	15.0 ± 3.0	17.0 ± 2.0	17.0 ± 2.64	15.0 ± 2.3
	160	15.0 ± 3.6	15.0 ± 1.0	17.0 ± 1.73	17.0 ± 1.0	16.0 ± 3.0
Ethyl acetate	10	12.0 ± 1.73	11.0 ± 1.73	13.0 ± 1.73	11.0 ± 1.0	15.0 ± 3.6
	20	12.0 ± 1.0	11.0 ± 1.0	12.0 ± 1.0	11.0 ± 1.0	9.0 ± 7.80
	40	13.0 ± 1.0	12.0 ± 2.0	14.0 ± 1.0	13.0 ± 1.0	14.0 ± 1.0
	80	14.0 ± 1.0	15.0 ± 1.73	15.0 ± 1.73	15.0 ± 1.73	15.0 ± 0
	160	16.0 ± 1.0	18.0 ± 1.0	17.0 ± 1.0	16.0 ± 2.0	17.0 ± 1.0
Methanol	10	10.0 ± 1.73	11.0 ± 1.0	12.0 ± 1.0	–	7.0 ± 6.08
	20	8.0 ± 7.0	11.0 ± 1.0	13.0 ± 3.0	–	14.0 ± 2.64
	40	12.0 ± 0	12.0 ± 1.0	13.0 ± 1.73	–	13.0 ± 1.0
	80	13.0 ± 1.0	13.0 ± 1.73	13.0 ± 1.0	–	14.0 ± 1.0
	160	14.0 ± 1.0	14.0 ± 1.0	16.0 ± 1.0	–	17.0 ± 3.6
Positive control	10 μg disc	29.6 ± 1.52	31.0 ± 1.0	30.3 ± 1.52	30.6 ± 0.57	30.3 ± 1.52

Data are means of three replicates (*n* = 3) ± standard deviation.

**TABLE 6:** Minimum inhibitory concentration (MIC) of the most effective plant extract against test organisms.

S. No.	Bacterial strain	MIC (mg mL^–1^)
1.	Escherichia coli*	64
2.	Pseudomonas aeruginosa*	8
3.	Klebsiella pneumoniae*	8
4.	Salmonella typhi*	32
5.	Staphylococcus aureus**	4

*Gram-negative bacteria. **Gram-positive bacteria.

## Discussion

In the present study, the analysis of *n*-hexane, ethyl acetate, and methanol root extracts of *S. anquetilia* revealed the existence of different bioactive principles, namely flavones, coumarins, glycosides, alkenes, carboxylic acids, tannins, phenols, amines, alkaloids, steroids, ketones, terpenoids, sesquiterpenes, fatty acid esters and alcohols, phyto-sterols, diterpenes, triterpene, etc. The therapeutic potential of different extracts of *S. anquetilia* may be attributed to the presence of these bioactive phytoconstituents. The FTIR analysis of *S. anquetilia* root extracts have shown several peaks signifying the presence of different functional groups in the extracts. The absence of absorption peak at 3,000–3,500 cm^–1^ in the IR spectrum of *n*-hexane and ethyl acetate extracts predicted the presence of a hydroxyl group (OH^–^). The major peaks that appeared in the extracts indicated the existence of functional groups such as alcohols, carboxylic acids, alkanes, aldehydes, amines, tertiary alcohols, aromatic compounds, aliphatic ketones, alkynes, etc. The FTIR analysis of petroleum ether seed oil extract of *Ziziphus spina-christi* revealed the presence of alcohols, phenols, alkanes, alkenes, carbonyls, carboxylic acids, and aromatic compounds as major functional groups (Abubaker et al., 2021). Visveshwari et al. (2017) while analyzing the methanol extract of *Ceropegia juncea* revealed the presence of alcohols, aldehydes, alkynes, alkenes, esters, and amines groups. These functional groups confirmed that *S. anquetilia* comprises a range of pharmaceutically significant bioactive constituents. The GC-MS investigation of root extracts of *S. anquetilia* showed the existence of 88 phyto-compounds in *n*-hexane, 74 phyto-compounds in ethyl acetate, and 112 phyto-compounds in methanol extracts, which add to the therapeutic values of this plant species. Some of the various bioactive compounds identified from the root extracts of *S. anquetilia* are known to possess significant biological activities. For instance, squalene is a triterpene and has been revealed to exhibit antitumor and antioxidant activities (Katerere et al., 2003; Amarowicz, 2009; Ganesh and Mohankumar, 2017), antimicrobial, and anticancer activity towards lung, skin, and colon oncogenesis (Rao et al., 1998; Smith, 2000). It also possesses anticancerous, gastro-preventive, hepato-protective, and pesticidal properties (Ukiva et al., 2002; Ganesh and Mohankumar, 2017), and as an anti-inflammatory, immune-stimulant, and lipogenase inhibitor (Yamuna et al., 2017). The *n*-hexadecanoic acid is a fatty acid, possesses antimicrobial, antioxidant, antiatherosclerotic (Cho et al., 2010), antiandrogenic (Komansilan et al., 2012), anticancer (Sabithira and Udayakumar, 2017), and antitumor activities. The *n*-hexadecanoic acid identified in plant extracts of *Benincasa hispida*, *Carissa congesta*, *Allium nigrum*, *Kielmeyera coriacea*, *Cyrtocarpa procera*, *Labisia pumila*, and *Rosa indica* and has been reported to possess antibacterial activity (Mickymaray et al., 2016). Also, using the *in-silico* method, the *n*-hexadecanoic acid has been used to design inhibitors specific to phospholipase A (2) by comparing with other known inhibitors as anti-inflammatory agents (Aparna et al., 2012; Qureshi et al., 2016). Furthermore, hexadecanoic acid, methyl ester is suggested to be a fatty acid and is reported to have antibacterial, antioxidant, nematicide, insecticide properties and also helps in lowering cholesterol.

Further, it possesses anticoronary, hepato-protective, antiacne, anti-inflammatory, antiarthritic, anticancer, antihistaminic, antieczemic, α-reductase inhibitor, and antiandrogenic activities (Krishnamoorthy and Subramaniam, 2014). Hexadecanoic acid, methyl ester has been shown to be a potent antibacterial agent against *S. aureus* W35, *P. aeruginosa* D31, *K. pneumoniae* DF30, and *K. pneumoniae* B45 strains (Shaaban et al., 2021). Lalthanpuii and Lalchhandama (2019) identified hexadecanoic acid, methyl ester as a major compound in *Imperata cylindrica* and reported its antibacterial properties against bacterial strains including *P. aeruginosa*, *Bacillus subtilis*, and *K. pneumoniae*. Various species use the antibacterial actions of fatty acid methyl ester to defend themselves against bacterial infections. Its principal focus of action is on bacterial cell membranes. It also disrupts cellular energy generation, impairs enzyme activity, and finally results in direct bacterial cell lysis. Owing to its safety and efficacy, it is a promising antibacterial therapeutic agent (Shaaban et al., 2021). (*Z*) 6, (*Z*) 9-pentadecadien-1-ol is fatty acid alcohol and possesses antibacterial activity (Sabithira and Udayakumar, 2017). 2R-acetoxymethyl-1,3,3-trimethyl-4t-(3-methyl-2-buten-1-yl)-1t-cyclohexanol is known to exhibit anticancer potential (Naine et al., 2016), anti-inflammatory, and antibacterial activities (Saravanan and Kasisankar, 2013). 1-hexyl-2-nitrocyclohexane is a ketone and exhibits antimicrobial (Al-Wathnani et al., 2012), and anti-inflammatory actions (Sivakumar and Gayathri, 2011; Ravisankar and Ester, 2017). 1-methylene-2b-hydroxymethyl-3,3-dimethyl-4b-(3-methyl but-2-enyl)-cyclohexane is sesquiterpene alcohol and it may act as an antimicrobial, anti-inflammatory, and anti-hyperlipidemic agent. 7H-furo[3,2-g][1]benzopyran-7-one,4,9-dimethoxy- also known as isopimpinellin is a furano-coumarin and it possesses antibacterial, antifungal, antibiofilm, and antioxidant potential (AlMalki, 2016).

Isopimpinellin, isolated from the hexane extract of *Peucedanum zenkeri* seeds, exhibited antibacterial property against *Cryptococcus neoformans* and *Mycobacterium intracellulare* (Ngunde Ngwendson et al., 2003; Mbah et al., 2010). In addition, it has shown significant insulin-stimulated lipogenesis inhibition, suggesting that it may trigger the lipolytic hormonal behavior and specifically reduces the antilipolytic hormonal effects (Kimura et al., 1982). Furthermore, it demonstrated mild cytotoxicity 39.2 μg mL^–1^ (IC50 value) to Colo-205 (Yang et al., 2003). 7-hydroxycoumarin also known as umbelliferone is a hydroxy-coumarin and it has been found to have several bioactivities *viz.*, antibacterial (Farshori et al., 2011), anticancer, and antidiabetic particularly type-2 diabetes mellitus (Ofentse, 2017). 7-hydroxyl-coumarin derivatives demonstrated effective antifungal and antibacterial activity against bacterial strains such as *B. subtilis*, *S. aureus*, *Streptococcus pyogene*, *P. aeruginosa*, *Salmonella typhimurium*, *E. coli*, and fungal strains of *Candida albicans*, *Candida krusei*, *Candida parapsilosis*, and *C. neoformans* (Farshori et al., 2011). 1,3,3-trimethyl-2-hydroxymethyl-3,3-dimethyl-4-(3-methyl but-2-enyl)-cyclohexene exhibits antibacterial and antioxidant activities (Sen et al., 2015). In this study, the root extracts of *S. anquetilia* exhibited remarkable *in-vitro* antibacterial potential towards the gram-positive and gram-negative bacterial strains by inhibiting their colony and growth rate, which is the first report from this study. Recently, Nabi et al. (2022a) reported the antibacterial potential of methanol leaf extract of *S. anquetilia* against *P. aeruginosa*, *E. coli*, *K. pneumoniae*, *S. typhi*, and *S. aureus*. Previously, the antimicrobial activity using the essential oil extracted from *S. laureola* was documented (Jangwan et al., 2010; Irshad et al., 2012). Similarly, Zeb et al. (2016) reported the antibacterial activity of aqueous leaf extract of *S. laureola*. It can be interpreted from the aforementioned justification that *S. anquetilia* possesses a wide range of therapeutic phytoconstituents capable of an array of bioactivities such as antibacterial, antifungal, antioxidant, anti-inflammatory, antidiabetic, antiaging, anticancer, hepato-protective, hypercholesterolemic, antihistaminic, anticoagulant, diuretic, etc. Therefore, the detection of different phyto-constituents of *n*-hexane, ethyl acetate, and methanol root extracts of *S. anquetilia* exhibits important pharmacological applications. Besides, investigations such as bioprospecting are required to sustain its pharmacological attributes and the biological significance of these novel bioconstituents will be noteworthy to be explored.

## Conclusion

The present study is the first report on the identification of various bioactive compounds by GC-MS analysis and antibacterial efficacy of root extracts of *S. anquetilia*. 5, 10-pentadecadien-1-ol, (*Z,Z*)- in *n*-hexane and ethyl acetate extracts, and 1-hexyl-2 nitrocyclohexane in methanol extract were the major compounds, respectively. These compounds may be responsible for the different therapeutic and pharmacological properties of *S. anquetilia* in conventional medicine. The root extracts of *S. anquetilia* demonstrated promising antibacterial efficacy towards the gram-positive as well as gram-negative bacterial strains as is obvious by high inhibition-zones, and lower MICs. This study, therefore, recommends the assessment of antibacterial activity of bioactive compounds from root extracts of *S. anquetilia*, which could be a vital source for the development of novel antibacterial drug candidate beneficial in the management of life-threatening microbial infections.

## Data availability statement

The original contributions presented in this study are included in the article/Supplementary material, further inquiries can be directed to the corresponding author.

## Author contributions

MN: conceptualization, methodology, data curation, formal analysis, software, and writing—original draft, review, and editing. NT: resources, investigation, and supervision. BAG: resources, investigation, supervision, and review. All authors contributed to the article and approved the submitted version.

## Abbreviations

MDR: multidrug-resistant
FTIR: fourier transform infrared spectrometer
GC-MS: gas chromatography-mass spectrometer
MIC: minimum inhibitory concentration
WHO: world health organization
NIST: national institute of standards and technology
MTCC: microbial type culture collection
MHB: mueller hinton broth
DMSO: dimethyl sulfoxide
ANOVA: analysis of variance.

## References

[B1] Abubaker M. A. Mohammed A. A. Farah A. A. Zhang J. (2021). Phytochemical screening by using GC-MS and FTIR spectrum analysis of fixed oil from Sudanese *Ziziphus spina christi* seeds. *Eurasian Chem. Commun.* 3 244–256. 10.22034/ecc.2021.273055.1137

[B2] AlMalki M. A. (2016). *In-vitro* antibacterial, antifungal, antibiofilm, and antioxidant potentials of isopimpinellin recovered from *Citrullus colocynthis*. *Int. J. Pharm. Pharm. Sci.* 8 117–122.

[B3] Al-Wathnani H. Ara I. Tahmaz R. R. Al-Dayel T. H. Bakir M. A. (2012). Bioactivity of natural compounds isolated from cyanobacteria and green algae against human pathogenic bacteria and yeast. *J. Med. Plants Res.* 6 3425–3433. 10.5897/JMPR11.1746

[B4] Amarowicz R. (2009). Squalene: a natural antioxidant? *Eur. J. Lipid Sci. Technol.* 111 411–412. 10.1002/ejlt.200900102

[B5] Aparna V. Dileep K. V. Mandal P. K. Karthe P. Sadasivan C. Haridas M. (2012). Anti-inflammatory property of n-hexadecanoic acid: structural evidence and kinetic assessment. *Chem. Biol. Drug Des.* 80 434–439. 10.1111/j.1747-0285.2012.01418.x 22642495

[B6] Banaras S. Javaid A. Khan I. H. (2020). Potential antifungal constituents of *Sonchus oleraceous* against *Macrophomina phaseolina*. *Int. J. Agric. Biol.* 24 1376–1382. 10.17957/IJAB/15.1573

[B7] Banaras S. Javaid A. Khan I. H. (2021). Bioassays guided fractionation of *Ageratum conyzoides* extract for the identification of natural antifungal compounds against *Macrophomina phaseolina*. *Int. J. Agric. Biol.* 25 761–767. 10.17957/IJAB/15.1727

[B8] Benarba B. Pandiella A. (2020). Medicinal plants as sources of active molecules against COVID-19. *Front. Pharmacol.* 11:1189. 10.3389/fphar.2020.01189 32848790 PMC7427466

[B9] Chakraborty B. Kumar R. S. Almansour A. I. Perumal K. Nayaka S. Brindhadevi K. (2022). *Streptomyces filamentosus* strain KS17 isolated from microbiologically unexplored marine ecosystems exhibited a broad spectrum of antimicrobial activity against human pathogens. *Process Biochem.* 117 42–52. 10.1016/j.procbio.2022.03.010

[B10] Cho K. H. Hong J. H. Lee K. T. (2010). Monoacylglycerol (MAG)-oleic acid has stronger antioxidant, anti-atherosclerotic, and protein glycation inhibitory activities than MAG-palmitic acid. *J. Med. Food* 13 99–107. 10.1089/jmf.2009.1024 20136442

[B11] Clinical and Laboratory Standards Institute (2008). *Clinical and Laboratory Standards Institute (CLSI) Methods for Dilution Antimicrobial Susceptibility Tests. Approved Standard, CLSI Document M7-A4*, 4th Edn. Wayne, PA: Clinical and Laboratory Standards Institute.

[B12] Dordab T. Sourinejad I. Nazemi M. (2021). Antibacterial effect of the squalene extracted from the liver of the Persian Gulf spot tail shark *Carcharhinus sorrah* (Müller & Henle, 1839). *Fish. Sci. Technol.* 10 251–258. Available online at: http://jfst.modares.ac.ir/article-6-50441-en.html

[B13] Farshori N. N. Banday M. R. Ahmad A. Khan A. U. Rauf A. (2011). 7-Hydroxy-coumarin derivatives: synthesis, characterization and preliminary antimicrobial activities. *Med. Chem. Res.* 20 535–541. 10.1007/s00044-010-9347-9

[B14] Ganesh M. Mohankumar M. (2017). Extraction and identification of bioactive components in *Sida cordata* (Burm. f.) using gas chromatography–mass spectrometry. *J. Food Sci. Technol.* 54 3082–3091. 10.1007/s13197-017-2744-z 28974793 PMC5602971

[B15] Gohar Y. M. El-Naggar M. M. Soliman M. K. Barakat K. M. (2010). Characterization of marine *Burkholderia cepacia* antibacterial agents. *J. Nat. Prod.* 3 86–94.

[B16] Gondwal M. Prakash O. Vivekanand, Pant A. K. Padalia R. C. Mathela C. S. (2012a). Essential oil composition and antioxidant activity of leaves and flowers of *Skimmia anquetilia* N.P. Taylor & Airy Shaw. *J. Essent. Oil Res.* 24 83–90. 10.1080/10412905.2012.646034

[B17] Gondwal M. Prakash O. Punetha H. Kanaujia S. Pant A. K. (2012b). Effect of essential oils of *Skimmia anquetilia* N.P. Taylor & Airy Shaw on fecundity, growth and development of *Caryedon serratus*. *Int. J. Biol. Pharm. Allied Sci.* 1 124–132.

[B18] Guo F. Liang Q. Zhang M. Chen W. Chen H. Yun Y. (2021). Antibacterial activity and mechanism of linalool against *Shewanella putrefaciens*. *Molecules* 26:245. 10.3390/molecules26010245 33466475 PMC7796449

[B19] Irshad M. Aziz S. Shahid M. Ahmed M. N. Minhas F. A. Sherazi T. (2012). Antioxidant and antimicrobial activities of essential oil of *Skimmea laureola* growing wild in the State of Jammu and Kashmir. *J. Med. Plants Res.* 6 1680–1684. 10.5897/JMPR11.1470

[B20] Jangwan J. S. Kumar N. Singh R. (2010). Analysis of composition and antibacterial activity of essential oil of *Skimmia lauriola* from Garhwal, Himalaya. *Int. J. Chem. Sci.* 8 1433–1439.

[B21] Javaid A. Naqvi S. F. Khan I. H. (2021). Ethyl acetate extract of *Chenopodium murale* root, a source of bioactive compounds. *Pak. J. Weed Sci. Res.* 27:93. 10.28941/pjwsr.v27i1.926

[B22] Javed S. Mahmood Z. Khan K. M. Sarker S. D. Javaid A. Khan I. H. (2021). Lupeol acetate as a potent antifungal compound against opportunistic human and phytopathogenic mold *Macrophomina phaseolina*. *Sci. Rep.* 11:8417. 10.1038/s41598-021-87725-7 33875698 PMC8055904

[B23] John P. Ahmad I. Aziz-ur-Rehman, Riaz T. Abbasi M. A. (2014). In-vitro evaluation of antioxidant activity of *Skimmia anquetilia* leaves extracts. *Int. Res. J. Pharm.* 5 143–150. 10.7897/2230-8407.050330

[B24] Katerere D. R. Gray A. I. Nash R. J. Waigh R. D. (2003). Antimicrobial activity of pentacyclic triterpenes isolated from African Combretaceae. *Phytochemistry* 63 81–88. 10.1016/S0031-9422(02)00726-4 12657301

[B25] Kimura Y. Ohminami H. Arichi H. Okuda H. Baba K. Kozawa M. (1982). Effects of various coumarins from roots of *Angelica dahurica* on actions of adrenaline, ACTH and insulin in fat cells. *Planta Med.* 45 183–187. 10.1055/s-2007-971370 6287510

[B26] Komansilan A. Abadi A. L. Yanuwiadi B. Kaligis D. A. (2012). Isolation and identification of biolarvicide from soursop (*Annona muricata* Linn) seeds to mosquito (*Aedes aegypti*) larvae. *Int. J. Eng. Technol.* 12 28–32.

[B27] Konappa N. Udayashankar A. C. Krishnamurthy S. Pradeep C. K. Chowdappa S. Jogaiah S. (2020). GC–MS analysis of phytoconstituents from *Amomum nilgiricum* and molecular docking interactions of bioactive serverogenin acetate with target proteins. *Sci. Rep.* 10:16438. 10.1038/s41598-020-73442-0 33009462 PMC7532471

[B28] Koparde A. A. Doijad R. C. Magdum C. S. (2019). “Natural products in drug discovery,” in *Pharmacognosy-Medicinal Plants*, eds Perveen S. Al-Taweel A. (London: IntechOpen), 1–20. 10.5772/intechopen.82860

[B29] Krishnamoorthy K. Subramaniam P. (2014). Phytochemical profiling of leaf, stem, and tuber parts of *Solena amplexicaulis* (Lam.) Gandhi using GC-MS. *Int. Sch. Res. Notices* 2014:567409. 10.1155/2014/567409 27379314 PMC4897340

[B30] Kumar V. Bhat Z. A. Kumar D. Khan N. A. Chashoo I. A. (2012). Evaluation of anti-inflammatory potential of leaf extracts of *Skimmia anquetilia*. *Asian Pac. J. Trop. Biomed.* 2 627–630. 10.1016/S2221-1691(12)60109-9 23569983 PMC3609364

[B31] Lalthanpuii P. B. Lalchhandama K. (2019). Chemical profiling, antibacterial and antiparasitic studies of *Imperata cylindrica*. *J. Appl. Pharm. Sci.* 9 117–121. 10.7324/JAPS.2019.91216

[B32] Li W. R. Shi Q. S. Liang Q. Xie X. B. Huang X. M. Chen Y. B. (2014). Antibacterial activity and kinetics of *Litsea cubeba* oil on *Escherichia coli*. *PLoS One* 9:e110983. 10.1371/journal.pone.0110983 25372706 PMC4220960

[B33] Lira M. H. P. D. Andrade Júnior F. P. D. Moraes G. F. Q. Macena G. D. S. Pereira F. D. O. Lima I. O. (2020). Antimicrobial activity of geraniol: an integrative review. *J. Essent. Oil Res.* 32 187–197. 10.1080/10412905.2020.1745697

[B34] Lisa S. R. Islam M. K. Qais N. (2020). Plants and plant constituents with analgesic and anti-inflammatory activities: a systematic review. *Dhaka Univ. J. Pharm. Sci.* 19 207–224. 10.3329/dujps.v19i2.50638

[B35] Liu X. Cai J. Chen H. Zhong Q. Hou Y. Chen W. (2020). Antibacterial activity and mechanism of linalool against *Pseudomonas aeruginosa*. *Microb. Pathog.* 141:103980. 10.1016/j.micpath.2020.103980 31962183

[B36] Liu Y. T. Chuang Y. C. Lo Y. S. Lin C. C. Hsi Y. T. Hsieh M. J. (2020). Asiatic acid, extracted from *Centella asiatica* and induces apoptosis pathway through the phosphorylation p38 mitogen-activated protein kinase in cisplatin-resistant nasopharyngeal carcinoma cells. *Biomolecules* 10:184. 10.3390/biom10020184 31991751 PMC7072674

[B37] Majeed M. M. Alashoor A. S. Alaoui-Sosse B. (2021). The antimicrobial effects of Chlorella vulgaris extracts on pathogenic bacteria isolated from burn patients. *Ann. Rom. Soc. Cell Biol.* 25, 17001–17009.

[B38] Mazurek B. Chmiel M. Górecka B. (2017). Fatty acids analysis using gas chromatography-mass spectrometer detector (GC/MSD)-method validation based on berry seed extract samples. *Food Anal. Methods* 10 2868–2880. 10.1007/s12161-017-0834-1

[B39] Mbah J. A. Gatsing D. Efange S. M. (2010). Antibacterial agents from the seeds of *Peucedanum zenkeri* L. (Umbelliferae). *Pak. J. Med. Sci.* 26 314–318.

[B40] Mickymaray S. Al Aboody M. S. Rath P. K. Annamalai P. Nooruddin T. (2016). Screening and antibacterial efficacy of selected Indian medicinal plants. *Asian Pac. J. Trop. Biomed.* 6 185–191. 10.1016/j.apjtb.2015.12.005

[B41] Nabi M. Tabassum N. Ganai B. A. (2022b). *Skimmia anquetilia* N.P. Taylor and Airy Shaw (Rutaceae): A critical appraisal of its ethnobotanical and pharmacological activities. *Front. Plant Sci.* 13:930687. 10.3389/fpls.2022.930687 35979070 PMC9377273

[B42] Nabi M. Zargar M. I. Tabassum N. Ganai B. A. Wani S. U. D. Alshehri S. (2022a). Phytochemical profiling and antibacterial activity of methanol leaf extract of *Skimmia anquetilia*. *Plants* 11:1667. 10.3390/plants11131667 35807619 PMC9268939

[B43] Naine S. J. Devi C. S. Mohanasrinivasan V. Doss C. Kumar D. T. (2016). Binding and molecular dynamic studies of sesquiterpenes (2R-acetoxymethyl-1, 3, 3-trimethyl-4t-(3-methyl-2-buten-1-yl)-1t-cyclohexanol) derived from marine *Streptomyces* sp. VITJS8 as potential anticancer agent. *Appl. Microbiol. Biotechnol.* 100 2869–2882. 10.1007/s00253-015-7156-2 26590587

[B44] Naqvi S. F. Khan I. H. Javaid A. (2020). Hexane soluble bioactive components of *Chenopodium murale* stem. *Pak. J. Weed Sci. Res.* 26 425–432. 10.28941/pjwsr.v26i4.875

[B45] Ngunde Ngwendson J. Bedir E. Efange S. M. N. Okunji C. O. Iwu M. M. Schuster B. G. (2003). Constituents of *Peucedanum zenkeri* seeds and their antimicrobial effects. *Die Pharmazie* 58 587–589. 12967040

[B46] Ofentse M. (2017). Umbelliferone: source, chemistry and bioactive review. *Bull. Fac. Pharm. Cairo Univ.* 55 223–232. 10.1016/j.bfopcu.2017.05.001

[B47] Oh H. N. Lee M. H. Kim E. Kwak A. W. Yoon G. Cho S. S. (2020). Licochalcone D induces ROS-dependent apoptosis in gefitinib-sensitive or resistant lung cancer cells by targeting EGFR and MET. *Biomolecules* 10:297. 10.3390/biom10020297 32070026 PMC7072161

[B48] O’Neil J. (2014). The Review on Antibiotic Resistance. Antimicrobial Resistance: Tackling a Crisis for the Health and Wealth of Nations, 1–16. Available online at: https://iiif.wellcomecollection.org/file/b28552179_AMR%20Tackling%20a%20crisis%20for%20the%20health%20and%20wealth%20of%20nations.pdf

[B49] Ouf S. A. Ali M. I. Haggag M. G. Elsafty D. O. Faraag A. H. (2022). Enhancement of antidermatophytic activities of *Citrullus colocynthis* Schrad collected from different ecological habitats in Egypt using fluconazole. *Phytomed. Plus* 2:100178. 10.1016/j.phyplu.2021.100178

[B50] Pan S. Y. Zhou S. F. Gao S. H. Yu Z. L. Zhang S. F. Tang M. K. (2013). New perspectives on how to discover drugs from herbal medicines: CAM’s outstanding contribution to modern therapeutics. *Evid. Based Complement. Alternat. Med.* 2013:627375. 10.1155/2013/627375 23634172 PMC3619623

[B51] Prakash O. Gondwal M. Pant A. K. (2011). Essential oils composition and antioxidant activity of water extract from seeds and fruit pulp of *Skimmia anquetilia* N.P. Taylor & Airy Shaw. *Indian J. Nat. Prod. Resour.* 2 435–441.

[B52] Qureshi T. Memon N. Memon S. Q. Ashraf M. A. (2016). Decontamination of ofloxacin: optimization of removal process onto sawdust using response surface methodology. *Desalination Water Treat.* 57 221–229. 10.1080/19443994.2015.1006825

[B53] Ranjith D. (2019). Molecular docking studies of aloe vera for their potential antibacterial activity using Argus lab 4.0. 1. *Pharma Innov. J.* 8 481–487.

[B54] Rao C. V. Newmark H. L. Reddy B. S. (1998). Chemopreventive effect of squalene on colon cancer. *Carcinogenesis* 19 287–290. 10.1093/carcin/19.2.287 9498278

[B55] Ravisankar M. Ester V. C. J. (2017). Determination of bioactive compounds in *Hydrophila auriculata* leaf extract using GC MS technique. *Int. J. Chem. Stud.* 5 729–733.

[B56] Reardon S. (2014). Antibiotic resistance sweeping developing world: bacteria are increasingly dodging extermination as drug availability outpaces regulation. *Nature* 509 141–143. 10.1038/509141a 24805322

[B57] Sabithira G. Udayakumar R. (2017). GC-MS analysis of methanolic extracts of leaf and stem of *Marsilea minuta* (Linn.). *J. Complement. Alternat. Med. Res.* 3 1–13. 10.9734/JOCAMR/2017/30871

[B58] Saravanan D. Kasisankar V. (2013). Asharani IV. GC-MS analysis of phytocomponents in the leaves of *Actinodaphne madraspatana* Bedd. *Int. J. Res. Pharm. Sci.* 4 469–473.

[B59] Sen S. Chakraborty R. (2015). Toward the integration and advancement of herbal medicine: a focus on traditional Indian medicine. *Botanics* 5 33–44. 10.2147/BTAT.S66308

[B60] Sen S. Talukdar N. C. Khan M. (2015). A simple metabolite profiling approach reveals critical biomolecular linkages in fragrant agarwood oil production from *Aquilaria malaccensis*–a traditional agro-based industry in North East India. *Curr. Sci.* 108 63–71.

[B61] Shaaban M. T. Ghaly M. F. Fahmi S. M. (2021). Antibacterial activities of hexadecanoic acid methyl ester and green-synthesized silver nanoparticles against multidrug-resistant bacteria. *J. Basic Microbiol.* 61 557–568. 10.1002/jobm.202100061 33871873

[B62] Shoaib M. Israyilova A. A. Ganbarov K. (2019). Cyclohexane and its functionally substituted derivatives: important class of organic compounds with potential antimicrobial activities. *J. Microbiol. Biotechnol. Food Sci.* 9 84–87. 10.15414/jmbfs.2019.9.1.84-87

[B63] Sim S. F. Lee T. Z. E. Mohd Irwan Lu N. A. L. Samling B. (2014). Synchronized analysis of FTIR spectra and GCMS chromatograms for evaluation of the thermally degraded vegetable oils. *J. Anal. Methods Chem.* 2014:271970. 10.1155/2014/271970 24563804 PMC3915895

[B64] Sivakumar V. Gayathri G. (2011). GC-MS analysis of bioactive components from ethanol extract of *Andrographis paniculata*. *World J. Pharm. Pharm. Sci.* 4 2031–2039.

[B65] Smith T. J. (2000). Squalene: potential chemopreventive agent. *Expert Opin. Investig. Drugs* 9 1841–1848. 10.1517/13543784.9.8.1841 11060781

[B66] Süntar I. (2020). Importance of ethnopharmacological studies in drug discovery: role of medicinal plants. *Phytochem. Rev.* 19 1199–1209. 10.1007/s11101-019-09629-9

[B67] Tran N. Pham B. Le L. (2020). Bioactive compounds in anti-diabetic plants: from herbal medicine to modern drug discovery. *Biology* 9:252. 10.3390/biology9090252 32872226 PMC7563488

[B68] Ukiva M. Akihisa T. Tokuda H. Suzuki H. Mukainaka T. Ichiishi E. (2002). Constituents of Compositae plants: III. Antitumor promoting effects and cytotoxic activity against human cancer lines of triterpene diols and triols from edible *Chrysanthemum* flowers. *Cancer Lett.* 177 7–12. 10.1016/S0304-3835(01)00769-8 11809525

[B69] Umaru I. J. Badruddin F. A. Umaru H. A. (2019). Phytochemical screening of essential oils and antibacterial activity and antioxidant properties of *Barringtonia asiatica* (L) leaf extract. *Biochem. Res. Int.* 2019:7143989. 10.1155/2019/7143989 30891316 PMC6390260

[B70] Visveshwari M. Subbaiyan B. Thangapandian V. (2017). Phytochemical analysis, antibacterial activity, FTIR AND GCMS analysis of *Ceropegia juncea* Roxb. *Int. J. Pharmacogn. Phytochem. Res.* 9 914–920. 10.25258/phyto.v9i07.11155

[B71] Wani T. A. Kumar N. Khan J. Shah S. N. Chandra S. (2016). In-vitro cytotoxic activity of *Skimmia anquetilia* Taylor & Airy Shaw essential oils on various human cancer cell lines. *Int. J. Res. Pharm. Chem.* 6 89–94.

[B72] Ward P. I. (2008). Environmental health perspectives. *J. Cell Biol.* 116 15–30.

[B73] Wulandari L. Retnaningtyas Y. Lukman H. (2016). Analysis of flavonoid in medicinal plant extract using infrared spectroscopy and chemometrics. *J. Anal. Methods Chem.* 2016:4696803. 10.1155/2016/4696803 27529051 PMC4977382

[B74] Yamuna P. Abirami P. Vijayashalini P. Sharmila M. (2017). GC-MS analysis of bioactive compounds in the entire plant parts of ethanolic extract of *Gomphrena decumbens* Jacq. *J. Med. Plants Stud.* 5 31–37.

[B75] Yang L. L. Wang M. C. Chen L. G. Wang C. C. (2003). Cytotoxic activity of coumarins from the fruits of *Cnidium monnieri* on leukemia cell lines. *Planta Med.* 69 1091–1095. 10.1055/s-2003-45188 14750023

[B76] Zeb A. Ahmad S. Ullah F. Ayaz M. Sadiq A. (2016). Anti-nociceptive activity of ethnomedicinally important analgesic plant *Isodon rugosus* Wall. ex Benth: mechanistic study and identifications of bioactive compounds. *Front. Pharmacol.* 7:200. 10.3389/fphar.2016.00200 27458379 PMC4933699

