# Supplementary material for: Phytochemical screening and antibacterial activity of Skimmia anquetilia N.P. Taylor and Airy Shaw: A first study from Kashmir Himalaya
Source: Front Plant Sci. 2022 Aug 12;13:937946. doi: 10.3389/fpls.2022.937946 (PMC9412939; doi:10.3389/fpls.2022.937946)
Supplement: Supplementary file 1 [file Table_1.docx]

**Table S1. Bioactive compounds from the *n*-hexane root extract of *Skimmia anquetilia***

| **S. No.** | **Compounds** | **Retention time (min)** | **CAS Number** | **Peak Area (%)** | **Molecular weight (g/mol)** | **Chemical formula** |
| --- | --- | --- | --- | --- | --- | --- |
|  | Butane, 2,2,3-trimethyl- | 2.136 | 464-06-2 | 0.42 | 100.2 | C_7_H_16_ |
|  | Cyclopropane, 1-ethyl-2-methyl-, *cis*- | 2.325 | 19781-68-1 | 3.91 | 84.16 | C_6_H_12_ |
|  | Cyclohexane | 2.541 | 110-82-7 | 1.57 | 84.16 | C_6_H_12_ |
|  | 2-propenoic acid, butyl ester | 5.143 | 141-32-2 | 0.04 | 128.16 | C_7_H_12_O_2_ |
|  | Pentanoic acid | 6.449 | 109-52-4 | 0.10 | 102.13 | C_5_H_10_O_2_ |
|  | 1, 6-octadien-3-ol, 3,7-dimethyl-, formate | 11.324 | 115-99-1 | 0.18 | 182.25 | C_11_H_18_O_2_ |
|  | 2, 4-decadienal, (E, Z)- | 11.466 | 25152-83-4 | 0.04 | 152.23 | C_10_H_16_O |
|  | [**Benzene,1-methyl-4-(2-propenyl)-**](https://www.lookchem.com/product_Benzene-1-methyl-4--2-propen-1-yl--/8873610.html) | 11.547 | 3333-13-9 | 0.06 | 132.20 | C_10_H_12_ |
|  | Geranyl acetate | 12.313 | 105-87-3 | 0.11 | 196.28 | C_12_H_20_O_2_ |
|  | 3-methoxy-4-hydroxy mandelonitrile | 12.622 | 0 | 0.04 | 179.17 | C_9_H_9_NO_3_ |
|  | Cyclohexane, 1-(1-propynyl)- | 13.129 | 1655-05-6 | 1.55 | 120.19 | C_9_H_12_ |
|  | (E)-ß-famesene | 13.302 | 18794-84-8 | 0.45 | 204.35 | C_15_H_24_ |
|  | 3,4-nonadien-6-yne, 5-ethyl-3-methyl- | 13.462 | 61227-88-1 | 0.03 | 162.28 | C_12_H_18_ |
|  | (Z,Z)-a-farnesene | 13.853 | 0 | 0.15 | 204.35 | [**C_15_H_24_**](https://pubchem.ncbi.nlm.nih.gov/#query=C15H24) |
|  | 3-heptyne, 5-methyl- | 13.989 | 61228-09-9 | 0.12 | 110.19 | C_8_H_14_ |
|  | 2-phenyl-5,6-dihydro-1,4-oxathiine | 14.190 | 41803-45-6 | 0.08 | 178.25 | [C_10_H_10_OS](https://pubchem.ncbi.nlm.nih.gov/#query=C10H10OS) |
|  | 1,2-pentadiene, 4-methoxy-4-methyl- | 14.561 | 49833-91-2 | 0.28 | 112.17 | [C_7_H_12_O](https://pubchem.ncbi.nlm.nih.gov/#query=C7H12O) |
|  | trans-nerolidyl formate | 14.625 | 0 | 0.08 | 250.38 | [**C_16_H_26_O_2_**](https://pubchem.ncbi.nlm.nih.gov/#query=C16H26O2) |
|  | 1-phenyl-1,4-nonanedione | 14.690 | 117937-13-0 | 0.09 | 232.31 | C_15_H_20_O_2_ |
|  | Malic acid, dipropyl ester | 14.880 | 1587-17-3 | 0.08 | 218.25 | [C_10_H_18_O_5_](https://pubchem.ncbi.nlm.nih.gov/#query=C10H18O5) |
|  | (E)-1-methyl-4-(6-methylhept-5-en-2-ylidene) cyclohex-1-ene | 15.000 | 53585-13-0 | 0.07 | 204.35 | [C_15_H_24_](https://pubchem.ncbi.nlm.nih.gov/#query=C15H24) |
|  | 1-hexyl-1-nitrocyclohexane | 15.357 | 118252-09-8 | 0.11 | 213.32 | C_12_H_23_NO_2_ |
|  | 1,3-diphenylbuta-1,2-diene | 16.105 | 53544-89-1 | 0.14 | 206.28 | [C_16_H_14_](https://pubchem.ncbi.nlm.nih.gov/#query=C16H14) |
|  | 3,5-dimethoxy-7-methylnaphthalene-2-ol | 16.302 | 75628-97-6 | 0.16 | 218.25 | [C_13_H_14_O_3_](https://pubchem.ncbi.nlm.nih.gov/#query=C13H14O3) |
|  | 2-methyl-3-(3-methyl-but-2-enyl)-2-(4-methyl-pent-3-enyl)-oxetane | 16.431 | 0 | 1.27 | 222.37 | [C_15_H_26_O](https://pubchem.ncbi.nlm.nih.gov/#query=C15H26O) |
|  | Tetradecanoic acid | 16.799 | 544-63-8 | 0.31 | 228.37 | C_14_H_28_O_2_ |
|  | 2-quinolinecarboxylic acid, 4,6-dihydroxy- | 17.084 | 3778-29-8 | 0.14 | 205.17 | [C_10_H_7_NO_4_](https://pubchem.ncbi.nlm.nih.gov/#query=C10H7NO4) |
|  | Ambrial | 17.533 | 3243-36-5 | 0.69 | 234.37 | C_16_H_26_O |
|  | 1,3,3-trimethyl-2-hydroxymethyl-3,3-dimethyl-4-(3-methylbut-2-enyl)-cyclohexene | 17.666 | 0 | 0.94 | 222.37 | C_15_H_26_O |
|  | benzo[1,2-b:4,3-b']difuran | 17.754 | 210-79-7 | 0.24 | 158.15 | [C_10_H_6_O_2_](https://pubchem.ncbi.nlm.nih.gov/#query=C10H6O2) |
|  | Pentadecanoic acid | 17.836 | 1002-84-2 | 0.15 | 242.39 | C_15_H_30_O_2_ |
|  | Benzoic acid, 3-fluoro-,2-oxo-2-phenylethyl ester | 17.945 | 55153-23-6 | 0.05 | 258.24 | [C_15_H_11_FO_3_](https://pubchem.ncbi.nlm.nih.gov/#query=C15H11FO3) |
|  | Cyclohexanol, 4-ethenyl-4-methyl-3-(1-methylethenyl)-, (1a,3a,4)- | 18.064 | 56298-45-4 | 0.04 | 180.29 | [C_12_H_20_O](https://pubchem.ncbi.nlm.nih.gov/#query=C12H20O) |
|  | Z-2-dodecenol | 18.224 | 69064-36-4 | 0.03 | 184.31 | C_12_H_24_O |
|  | Tetradecanoic acid, 10, 13-dimethyl-, methyl ester | 18.489 | 267650-23-7 | 0.27 | 270.45 | C_17_H_34_O_2_ |
|  | 1-hexyl-2-nitrocyclohexane | 18.758 | 118252-04-3 | 4.35 | 213.32 | C_12_H_23_NO_2_ |
|  | *n*-hexadecanoic acid | 19.004 | 57-10-3 | 13.41 | 256.42 | C_16_H_32_O_2_ |
|  | Dihydro-*cis*-a-copaene-8-ol | 19.303 | 58569-27-0 | 1.78 | 222.36 | C_15_H_26_O |
|  | 2R-acetoxymethyl-1,3,3-trimethyl-4t-(3-methyl-2-buten-1-yl)-1t-cyclohexanol | 19.537 | 0 | 1.01 | 282.4 | [**C_17_H_30_O_3_**](https://pubchem.ncbi.nlm.nih.gov/#query=C17H30O3) |
|  | [**8a(2H)-phenanthrenol, 7-ethenyldodecahydro-1,1,4a,7-tetramethyl-, acetate, [4as-(4a. alpha.,4b. beta.,7. beta.,8a. alpha., 10a.beta.)]-**](https://www.ncbi.nlm.nih.gov/pcsubstance/?term=%228a(2H)-Phenanthrenol%2C%207-ethenyldodecahydro-1%2C1%2C4a%2C7-tetramethyl-%2C%20acetate%2C%20%5B4as-(4a.alpha.%2C4b.beta.%2C7.beta.%2C8a.alpha.%2C10a.beta.)%5D-%22%5bCompleteSynonym%5d%20AND%20534447%5bStandardizedCID%5d) | 19.622 | 41756-14-3 | 7.30 | 332.5 | [C_22_H_36_O_2_](https://pubchem.ncbi.nlm.nih.gov/#query=C22H36O2) |
|  | l-alanine, N-(3-trifluoromethylbenzoyl)-, hexyl ester | 19.842 | 0 | 4.02 | 345.36 | [C_17_H_22_F_3_NO_3_](https://pubchem.ncbi.nlm.nih.gov/#query=C17H22F3NO3) |
|  | 2,6,10,14-hexadecatetraenoic acid, 3,7,11,15-tetramethyl-9-(phenylsulfonyl)-, methyl ester, (E,E,E)- (9CI) | 19.992 | 74367-17-2 | 0.81 | 458.7 | [C_27_H_38_O_4_S](https://pubchem.ncbi.nlm.nih.gov/#query=C27H38O4S) |
|  | 7H-furo[3,2-g][1] benzopyran-7-one, 4-methoxy- | 20.108 | 484-20-8 | 1.82 | 216.18 | C_12_H_8_O_4_ |
|  | Phenanthrene, 9, 10-diethyl-3,6-dimethoxy- | 20.220 | 5025-38-7 | 1.36 | 294.4 | [C_20_H_22_O_2_](https://pubchem.ncbi.nlm.nih.gov/#query=C20H22O2) |
|  | 1,2-pentanediol,5-(6-bromodecahydro-2-hydroxy-2,5,5a,8a-tetramethyl-1-naphthalenyl)-3-methylene-, 1,2-diacetate | 20.288 | 115346-29-7 | 0.07 | 487.5 | [C_24_H_39_BrO_5_](https://pubchem.ncbi.nlm.nih.gov/#query=C24H39BrO5) |
|  | Butyl isobutyl isobutal | 20.384 | 0 | 0.25 | 202.33 | [C_12_H_26_O_2_](https://pubchem.ncbi.nlm.nih.gov/#query=C12H26O2) |
|  | 5, 10-pentadecadien-1-ol, (Z,Z)- | 20.821 | 64275-51-0 | 33.94 | 224.38 | [C_15_H_28_O](https://pubchem.ncbi.nlm.nih.gov/#query=C15H28O) |
|  | 5, 10-pentadecandiyn-1-ol | 21.326 | 64275-50-9 | 0.14 | 220.35 | [C_15_H_24_O](https://pubchem.ncbi.nlm.nih.gov/#query=C15H24O) |
|  | 2,3-dihydro-2-ethyl-1H-cyclopenta[b]quinoxaline | 21.421 | 109682-73-7 | 0.16 | 198.26 | [C_13_H_14_N_2_](https://pubchem.ncbi.nlm.nih.gov/#query=C13H14N2) |
|  | 2,2,3,3,4,4-hexamethyltetrahydrofuran | 21.537 | 0 | 0.09 | 156.26 | C_10_H_20_O |
|  | 7H-furo[3,2-g][1] benzopyran-7-one,4,9-dimethoxy- | 21.713 | 482-27-9 | 2.56 | 246.21 | C_13_H_10_O_5_ |
|  | N-methyl-2-tert, -butoxycarbonylazetidine | 21.788 | 51764-31-9 | 0.08 | 171.24 | [C_9_H_17_NO_2_](https://pubchem.ncbi.nlm.nih.gov/#query=C9H17NO2) |
|  | trans-4-tert-butylcycloheptanol | 21.928 | 6221-53-0 | 0.08 | 170.29 | [C_11_H_22_O](https://pubchem.ncbi.nlm.nih.gov/#query=C11H22O) |
|  | 1-methyl-4-isopropyl-cyclohexyl 2-hydroperfluorobutanoate | 21.992 | 0 | 0.07 | 334,3 | [C_14_H_20_F_6_O_2_](https://pubchem.ncbi.nlm.nih.gov/#query=C14H20F6O2) |
|  | 2,6,10-dedecatrien-1-ol,3,7,11-trimethyl-9-(phenylsulfonyl)-,(E,E)- | 22.077 | 57683-67-7 | 0.07 | 362.5 | [C_21_H_30_O_3_S](https://pubchem.ncbi.nlm.nih.gov/#query=C21H30O3S) |
|  | *cis*,*cis*- and *cis*,trans-1,9-dimethylspiro[4.5]decane | 22.173 | 0 | 0.22 | 166.30 | C_12_H_22_ |
|  | Isoauraptene | 22.387 | 1088-17-1 | 0.06 | 260.27 | [C_15_H_16_O_4_](https://pubchem.ncbi.nlm.nih.gov/#query=C15H16O4) |
|  | Heptanes, 3,4,5-trimethyl- | 22.604 | 20278-89-1 | 0.05 | 142.28 | [C_10_H_22_](https://pubchem.ncbi.nlm.nih.gov/#query=C10H22) |
|  | 1(2-thiophen-2-yl-vinylcarbonyl)-4-methoxy-benzene | 22.710 | 0 | 0.16 | 244.31 | [C_14_H_12_O_2_S](https://pubchem.ncbi.nlm.nih.gov/#query=C14H12O2S) |
|  | Methyl *cis*-cinnamate | 22.788 | 19713-73-6 | 0.04 | 162.18 | [C_10_H_10_O_2_](https://pubchem.ncbi.nlm.nih.gov/#query=C10H10O2) |
|  | 1-formyl-2,2,6-trimethyl-3-cis-(3-methylbut-2-enyl)-5-cyclohexene | 22.873 | 0 | 0.03 | 220.35 | [C_15_H_24_O](https://pubchem.ncbi.nlm.nih.gov/#query=C15H24O) |
|  | Eicosanal | 23.013 | 2400-66-0 | 0.07 | 296.5 | [C_20_H_40_O](https://pubchem.ncbi.nlm.nih.gov/#query=C20H40O) |
|  | Photocitral B | 23.188 | 6040-45-5 | 0.27 | 152.23 | C_10_H_16_O |
|  | 7H-furo(3,2-g)(1)benzopyran-7-one, 9-(2,3-epoxy-3-methylbut) | 23.421 | 2880-49-1 | 0.14 | 286.28 | [C_16_H_14_O_5_](https://pubchem.ncbi.nlm.nih.gov/#query=C16H14O5) |
|  | 2,6,10,14-tetramethylpentadecan-6-ol | 23.679 | 104000-14-8 | 0.25 | 284.5 | [C_19_H_40_O](https://pubchem.ncbi.nlm.nih.gov/#query=C19H40O) |
|  | 2,2-dimethyl-propyl 2,2-dimethyl-propanesulfinyl Sulfone | 24.002 | 82360-14-3 | 0.16 | 254.4 | [C_10_H_22_O_3_S_2_](https://pubchem.ncbi.nlm.nih.gov/#query=C10H22O3S2) |
|  | Pabulenol | 24.108 | 55297-82-0 | 0.89 | 286.28 | [**C_16_H_14_O_5_**](https://www.chemeo.com/search?q=C16H14O5) |
|  | Octadecane, 2-methyl- | 24.305 | 1560-88-9 | 0.09 | 268.5 | [C_19_H_40_](https://pubchem.ncbi.nlm.nih.gov/#query=C19H40) |
|  | Nonacos-1-ene | 24.390 | 18835-35-3 | 0.06 | 400.72 | C_29_H_52_ |
|  | Tetracosanal | 24.587 | 57866-08-7 | 0.05 | 352.6 | [C_24_H_48_O](https://pubchem.ncbi.nlm.nih.gov/#query=C24H48O) |
|  | 2H-1-benzopyran-2-one, 7-[(3,7-dimethyl-2,6-octadienyl) oxy]-, (E)- | 24.710 | 495-02-3 | 0.09 | 298.4 | [C_19_H_22_O_3_](https://pubchem.ncbi.nlm.nih.gov/#query=C19H22O3) |
|  | 10-heneicosene (c,t) | 25.046 | 95008-11-0 | 0.19 | 294.6 | [C_21_H_42_](https://pubchem.ncbi.nlm.nih.gov/#query=C21H42) |
|  | Methyl (2R, 3R, 4S)-3-(tert-butyldimethylsilyloxy)-2,4-dimethylhexanoate | 25.176 | 0 | 0.02 | 288.21 | [C_15_H_32_O_3_Si](https://pubchem.ncbi.nlm.nih.gov/#query=C15H13N3O) |
|  | (Z)-3,7-dimethylocta-2,6-dien-1-yl palmitate | 25.347 | 122569-17-9 | 0.32 | 392.65 | C_26_H_48_O_2_ |
|  | Ethanone, 1,1’-(6-methoxy-2,5-benzofurandiyl) bis- | 25.597 | 23840-15-5 | 0.30 | 232.23 | [C_13_H_12_O_4_](https://pubchem.ncbi.nlm.nih.gov/#query=C13H12O4) |
|  | 2H-1,3,4-benzotriazepin-2-one, 1,3-dihydro-7-methyl-5-phenyl- | 25.754 | 2855-57-4 | 0.13 | 251.28 | [C_15_H_13_N_3_O](https://pubchem.ncbi.nlm.nih.gov/#query=C15H13N3O) |
|  | Squalene | 26.060 | 111-02-4 | 3.59 | 410.72 | C_30_H_50_ |
|  | 3-buten-2-ol, 4-(2,2,6-trimethyl-7-oxabicyclo [4.1.0]hept-1-yl)- | 26.305 | 51138-08-0 | 0.07 | 210.31 | [C_13_H_22_O_2_](https://pubchem.ncbi.nlm.nih.gov/#query=C13H22O2) |
|  | Octadecane, 2-methyl- | 26.468 | 1560-88-9 | 0.23 | 268.5 | [C_19_H_40_](https://pubchem.ncbi.nlm.nih.gov/#query=C19H40) |
|  | 1-methylene-2b-hydroxymethyl-3,3-dimethyl-4b-(3-methylbut-2-enyl)-cyclohexane | 26.625 | 0 | 0.57 | 222.37 | [C_15_H_26_O](https://pubchem.ncbi.nlm.nih.gov/#query=C15H26O) |
|  | (9Z,12Z) -(E)-3,7-dimethylocta-2,6-dien-1-yl-octadeca-9,12-dienoate | 26.733 | 0 | 0.48 | 416.7 | [C_28_H_48_O_2_](https://pubchem.ncbi.nlm.nih.gov/#query=C28H48O2) |
|  | Cyclohexane, 1,5,5-trimethyl-6-(2-propenylidene)- | 27.750 | 56248-17-0 | 0.65 | 162.27 | [C_12_H_18_](https://pubchem.ncbi.nlm.nih.gov/#query=C12H18) |
|  | Borane, diethyl(decyloxy)- | 28.121 | 0 | 0.08 | 226.21 | [**C_14_H_31_BO**](https://pubchem.ncbi.nlm.nih.gov/#query=C14H31BO) |
|  | Benzene, 1,3,5-trimethyl-2-(1,2-propdienyl)- | 28.386 | 29555-07-5 | 0.45 | 158.24 | [C_12_H_14_](https://pubchem.ncbi.nlm.nih.gov/#query=C12H14) |
|  | Clionasterol acetate | 28.601 | 4651-54-1 | 0.67 | 456.7 | [C_31_H_52_O_2_](https://pubchem.ncbi.nlm.nih.gov/#query=C31H52O2) |
|  | dl-α-tocopherol | 28.733 | 10191-41-0 | 0.59 | 430.7 | [C_29_H_50_O_2_](https://pubchem.ncbi.nlm.nih.gov/#query=C29H50O2) |
|  | (2E,6E)-3,7,11-trimethyldodeca-2,6,10-trien-1-yl-dodecanoate | 29.281 | 78368-58-8 | 0.47 | 404.67 | [C_27_H_48_O_2_](https://pubchem.ncbi.nlm.nih.gov/#query=C29H50O2) |
|  | Campesterol | 30.070 | 474-62-4 | 1.30 | 400.7 | [C_28_H_48_O](https://pubchem.ncbi.nlm.nih.gov/#query=C28H48O) |
