# Supplementary material for: Phytochemical screening and antibacterial activity of Skimmia anquetilia N.P. Taylor and Airy Shaw: A first study from Kashmir Himalaya
Source: Front Plant Sci. 2022 Aug 12;13:937946. doi: 10.3389/fpls.2022.937946 (PMC9412939; doi:10.3389/fpls.2022.937946)
Supplement: Supplementary file 2 [file Table_2.docx]

**Table S2. Bioactive compounds from the ethyl acetate root extract of *Skimmia anquetilia***

| **S. No.** | **Compounds** | **Retention time (min)** | **CAS Number** | **Peak Area (%)** | **Molecular weight (g/mol)** | **Chemical formula** |
| --- | --- | --- | --- | --- | --- | --- |
|  | 2-propenoic acid, butyl ester | 5.153 | 141-32-2 | 0.26 | 128.16 | [C_7_H_12_O_2_](https://pubchem.ncbi.nlm.nih.gov/#query=C7H12O2) |
|  | Hexanoic acid | 6.367 | 142-62-1 | 0.22 | 116.16 | [C_6_H_12_O_2_](https://pubchem.ncbi.nlm.nih.gov/#query=C6H12O2) |
|  | Linalyl acetate | 10.598 | 115-95-7 | 0.43 | 196.29 | [C_12_H_20_O_2_](https://pubchem.ncbi.nlm.nih.gov/#query=C12H20O2) |
|  | 1,3-diacetin | 11.813 | 0 | 0.22 | 176.17 | [C_7_H_12_O_5_](https://pubchem.ncbi.nlm.nih.gov/#query=C7H12O5) |
|  | Geranyl acetate | 12.323 | 105-87-3 | 0.24 | 196.28 | C_12_H_20_O_2_ |
|  | Cyclohexene, 1-(1-propynyl)- | 12.770 | 1655-05-6 | 1.04 | 120.19 | C_9_H_12_ |
|  | Dihydro-*cis*- α-copaene-8-ol | 13.496 | 58569-27-0 | 0.82 | 222.36 | C_15_H_26_O |
|  | 3-heptyne, 5-methyl- | 14.003 | 61228-09-9 | 0.11 | 110.2 | [C_8_H_14_](https://pubchem.ncbi.nlm.nih.gov/#query=C8H14) |
|  | Dodecanoic acid | 14.517 | 143-07-7 | 0.16 | 200.32 | [C_12_H_24_O_2_](https://pubchem.ncbi.nlm.nih.gov/#query=C12H24O2) |
|  | 1,2-naphthalenedione, 6-hydroxy- | 14.894 | 607-20-5 | 0.15 | 174.15 | C_10_H_6_O_3_ |
|  | Tricycle [3.3.1.1(3,7)] decane-2,6-dione, 4-acetyl- | 16.118 | 56781-92-1 | 0.29 | 206.24 | [C_12_H_14_O_3_](https://pubchem.ncbi.nlm.nih.gov/#query=C12H14O3) |
|  | 1,15-pentadecanediol | 16.316 | 14722-40-8 | 0.46 | 244.41 | [C_15_H_32_O_2_](https://pubchem.ncbi.nlm.nih.gov/#query=C15H32O2) |
|  | t-butyl cyclopentane peroxy carboxylate | 16.761 | 25023-14-7 | 0.16 | 186.25 | [C_10_H_18_O_3_](https://pubchem.ncbi.nlm.nih.gov/#query=C10H18O3) |
|  | 2-quinolinecarboxylic acid, 4,6-dihydroxy- | 17.098 | 3778-29-8 | 0.25 | 205.17 | [C_10_H_7_NO_4_](https://pubchem.ncbi.nlm.nih.gov/#query=C10H7NO4) |
|  | Ambrial | 17.540 | 3243-36-5 | 0.92 | 234.37 | C_16_H_26_O |
|  | 1,3,3-trimethyl-2-hydroxymethyl-3,3-dimethyl-4-(3-methylbut-2-enyl)-cyclohexene | 17.673 | 0 | 4.65 | 222.37 | C_15_H_26_O |
|  | Benzo[1,2-b:4,3-b’] difuran | 17.761 | 210-79-7 | 0.51 | 158.15 | [C_10_H_6_O_2_](https://pubchem.ncbi.nlm.nih.gov/#query=C10H6O2) |
|  | Phthalic acid, hept-4-yl isobutyl ester | 18.003 | 0 | 0.59 | 320.4 | [C_19_H_28_O_4_](https://pubchem.ncbi.nlm.nih.gov/#query=C19H28O4) |
|  | Tetradecanoic acid, 10, 13-dimethyl-, methyl ester | 18.492 | 267650-23-7 | 0.62 | 270.45 | C_17_H_34_O_2_ |
|  | 2-dodecen-1-ol, 12-chloro- | 18.683 | 74810-78-9 | 0.55 | 218.76 | [C_12_H_23_ClO](https://pubchem.ncbi.nlm.nih.gov/#query=C12H23ClO) |
|  | *n*-hexadecanoic acid | 18.894 | 57-10-3 | 5.54 | 256.42 | C_16_H_32_O_2_ |
|  | 3,4-heptadien-2-one, 3-cyclopentyl-6-methyl- | 19.047 | 63922-50-9 | 0.42 | 192.3 | [C_13_H_20_O](https://pubchem.ncbi.nlm.nih.gov/#query=C13H20O) |
|  | Hexadecanoic acid, ethyl ester | 19.163 | 628-97-7 | 2.13 | 284.5 | [C_18_H_36_O_2_](https://pubchem.ncbi.nlm.nih.gov/#query=C18H36O2) |
|  | 5,6-decadien-3-yne, 5,7-diethyl- | 19.227 | 61227-89-2 | 0.74 | 190.32 | [C_14_H_22_](https://pubchem.ncbi.nlm.nih.gov/#query=C14H22) |
|  | (E)-15,16-dinorlabda-8(17), 11-dien-13-one | 19.377 | 76497-69-3 | 0.39 | 260.39 | [C_18_H_28_O](https://pubchem.ncbi.nlm.nih.gov/#query=C18H28O) |
|  | l-alanine, N-(3-trifluoromethylbenzoyl)-, isohexyl ester | 19.843 | 0 | 3.49 | 345.36 | [C_17_H_22_F_3_NO_3_](https://pubchem.ncbi.nlm.nih.gov/#query=C17H22F3NO3) |
|  | Cyclobutaneacetonitrile, 1-methyl-2-(1-methylethylidene)- | 19.986 | 55760-14-0 | 0.27 | 149.23 | [C_10_H_15_N](https://pubchem.ncbi.nlm.nih.gov/#query=C10H15N) |
|  | 7H-furo[3,2-g][1]benzopyran-7-one, 4-methoxy- | 20.057 | 484-20-8 | 1.12 | 216.18 | C_12_H_8_O_4_ |
|  | 9, 12-octadecadienoic acid, methyl ester | 20.145 | 2462-85-3 | 0.37 | 294.47 | C_19_H_34_O_2_ |
|  | Naphthalene, 1,7-dimethoxy- | 20.210 | 5309-18-2 | 1.13 | 188.22 | C_12_H_12_O_2_ |
|  | 2-(4-phenyl-1,3-butadienyl)-2-methyl-1,3-dioxolane | 20.445 | 80283-24-5 | 1.25 | 216.27 | [C_14_H_16_O_2_](https://pubchem.ncbi.nlm.nih.gov/#query=C14H16O2) |
|  | 5,10-pentadecadien-1-ol, (Z,Z)- | 20.618 | 64275-51-0 | 10.48 | 224.38 | [C_15_H_28_O](https://pubchem.ncbi.nlm.nih.gov/#query=C15H28O) |
|  | 9(E),11(E)-conjugated linoleic acid, ethyl ester | 20.758 | 0 | 2.80 | 280.5 | C_18_H_32_O_2_ |
|  | Ethyl 13-methyl-tetradecanoate | 21.006 | 0 | 0.44 | 270.5 | [C_17_H_34_O_2_](https://pubchem.ncbi.nlm.nih.gov/#query=C17H34O2) |
|  | (3E,5E,7E)-6-methyl-8-(2,6,6-trimethyl-1-cyclohexenyl)-3,5,7-octatrien-2-one | 21.196 | 17974-57-1 | 0.24 | 258.39 | [C_18_H_26_O](https://pubchem.ncbi.nlm.nih.gov/#query=C18H26O) |
|  | 2H-furo[2,3-h]-1-benzopyran-2-one-, 8-(1-methylethenyl)- | 21.387 | 1760-27-6 | 0.13 | 226.23 | [C_14_H_10_O_3_](https://pubchem.ncbi.nlm.nih.gov/#query=C14H10O3) |
|  | 2R-acetoxymethyl-1,3,3-trimethyl-4t-(3-methyl-2-buten-1-yl)-1t-cyclohexanol | 21.475 | 0 | 2.87 | 282.4 | [C_17_H_30_O_3_](https://pubchem.ncbi.nlm.nih.gov/#query=C17H30O3) |
|  | 7,7,8,8-tetramethylbicyclo[4.2.0]octa-1(6),3-diene-2,5-dione | 21.656 | 77627-51-1 | 2.84 | 190.24 | [C_12_H_14_O_2_](https://pubchem.ncbi.nlm.nih.gov/#query=C12H14O2) |
|  | Undecane, 2,6-dimethyl- | 21.917 | 17301-23-4 | 0.19 | 184.36 | [C_13_H_28_](https://pubchem.ncbi.nlm.nih.gov/#query=C13H28) |
|  | 5-thiazolidinone, 4-(2-furanylmethylene)-3-phenyl- | 22.057 | 54986-57-1 | 0.29 | 257.31 | [C_14_H_11_NO_2_S](https://pubchem.ncbi.nlm.nih.gov/#query=C14H11NO2S) |
|  | 1-methyl-4-isopropyl-cyclohexyl 2-hydroperfluorobutanoate | 22.166 | 0 | 1.04 | 334,3 | [C_14_H_20_F_6_O_2_](https://pubchem.ncbi.nlm.nih.gov/#query=C14H20F6O2) |
|  | 1,4-methanobiphenylen-9-Ol, 2-chloro-1,2,3,4,4a,8b-hexahydro-, (1α,2α,4α,4aα,8bα,9S*)- | 22.366 | 52021-31-5 | 0.05 | 220.69 | [C_13_H_13_ClO](https://pubchem.ncbi.nlm.nih.gov/#query=C13H13ClO) |
|  | 6-(3-methyl-3-cyclohexenyl)-2-methyl-2,6-heptadienol | 22.451 | 18681-09-9 | 0.16 | 220.35 | [C_15_H_24_O](https://pubchem.ncbi.nlm.nih.gov/#query=C15H24O) |
|  | 5, 10-pentadecadiyne, 1-chloro- | 22.881 | 64275-44-1 | 0.41 | 238.79 | [C_15_H_23_Cl](https://pubchem.ncbi.nlm.nih.gov/#query=C15H23Cl) |
|  | [10-heneicosene](https://pubchem.ncbi.nlm.nih.gov/compound/5364553)  (c,t) | 23.203 | 95008-11-0 | 0.17 | 294.6 | [C_21_H_42_](https://pubchem.ncbi.nlm.nih.gov/#query=C21H42) |
|  | (3E,5E,7E)-6-methyl-8-(2,6,6-trimethyl-1-cyclohexenyl)-3,5,7-octatrien-2-one | 23.394 | 17974-57-1 | 1.03 | 258.39 | [C_18_H_26_O](https://pubchem.ncbi.nlm.nih.gov/#query=C18H26O) |
|  | 1-heneicosanol | 23.496 | 15594-90-8 | 0.30 | 312.57 | C_21_H_44_O |
|  | Fern-7-en-3β-ol | 23.666 | 4966-00-1 | 0.62 | 426.7 | [C_30_H_50_O](https://pubchem.ncbi.nlm.nih.gov/#query=C30H50O) |
|  | 4-(4-fluorophenyl)-2,3-dihydro-2-methyl—1H-1,5-benzodiazepine | 23.764 | 111536-74-4 | 0.32 | 254.3 | [C_16_H_15_FN_2_](https://pubchem.ncbi.nlm.nih.gov/#query=C16H15FN2) |
|  | Phthalic acid, di(2-propylpentyl) ester | 24.006 | 0 | 7.41 | 390.6 | [C_24_H_38_O_4_](https://pubchem.ncbi.nlm.nih.gov/#query=C24H38O4) |
|  | 1-hexyl-1-nitrocyclohexane | 24.081 | 118252-09-8 | 0.43 | 213.32 | C_12_H_23_NO_2_ |
|  | Photocitral B | 24.217 | 6040-45-5 | 0.19 | 152.23 | C_10_H_16_O |
|  | Nonacos-1-ene | 24.337 | 18835-35-3 | 0.9 | 400.72 | C_29_H_52_ |
|  | Terephthalic acid, 3,4-dichlorophenyl undecyl ester | 24.519 | 0 | 0.27 | 465.4 | [C_25_H_30_Cl_2_O_4_](https://pubchem.ncbi.nlm.nih.gov/#query=C25H30Cl2O4) |
|  | 2H-1-benzopyran-2-one, 7-[(3,7 -dimethyl-2,6-octadienyl)oxy]-, (E)- | 24.713 | 495-02-3 | 0.23 | 298.4 | C_19_H_22_O_3_ |
|  | 1-hexyl-2-nitrocyclohexane | 24.922 | 118252-04-3 | 7.94 | 213.32 | C_12_H_23_NO_2_ |
|  | 2-isopropyl-5-methylcyclohexyl 3-(1-(4-chlorophenyl)-3-oxobutyl)-coumarin-4-yl carbonate | 25.121 | 0 | 2.57 | 525 | [C_30_H_33_ClO_6_](https://pubchem.ncbi.nlm.nih.gov/#query=C30H33ClO6) |
|  | (Z)-3,7-dimethylocta-2,6-dien-1-yl palmitate | 25.444 | 122569-17-9 | 0.17 | 392.65 | C_26_H_48_O_2_ |
|  | 3-ethyl-2,6,10-trimethylundecane | 25.768 | 0 | 0.32 | 226.44 | [C_16_H_34_](https://pubchem.ncbi.nlm.nih.gov/#query=C16H34) |
|  | Squalene | 26.050 | 111-02-4 | 3.79 | 410.72 | C_30_H_50_ |
|  | Sulfurous acid, butyl nonyl ester | 26.475 | 0 | 0.09 | 264.43 | [C_13_H_28_O_3_S](https://pubchem.ncbi.nlm.nih.gov/#query=C13H28O3S) |
|  | 2-cyclopropen-1-ol, 1,2-dicyclopentyl-3-(1-methylethyl)-, acetate | 26.662 | 69611-52-5 | 4.25 | 276.4 | [C_18_H_28_O_2_](https://pubchem.ncbi.nlm.nih.gov/#query=C18H28O2) |
|  | 6-tetradecane sulfonic acid, butyl ester | 27.244 | 0 | 0.17 | 334.6 | [C_18_H_38_O_3_S](https://pubchem.ncbi.nlm.nih.gov/#query=C18H38O3S) |
|  | 1-methylene-2b-hydroxymethyl-3,3-dimethyl-4b-(3-methylbut-2-enyl)-cyclohexane | 27.448 | 0 | 5.59 | 222.37 | [C_15_H_26_O](https://pubchem.ncbi.nlm.nih.gov/#query=C15H26O) |
|  | 5,9-dimethyl-2-(1-methylethylidene)-1-cyclodecanol | 27.580 | 69239-72-1 | 1.13 | 224.38 | [C_15_H_28_O](https://pubchem.ncbi.nlm.nih.gov/#query=C15H28O) |
|  | 1,2-pentanediol, 5-(6-bromodecahydro-2-hydroxy-2,5,5a,8a-tetramethyl-1-naphthalenyl)-3-methylene-, 1,2-diacetate | 27.645 | 115346-29-7 | 2.11 | 487.5 | [C_24_H_39_BrO_5_](https://pubchem.ncbi.nlm.nih.gov/#query=C24H39BrO5) |
|  | Campesterol, acetate | 27.754 | 0 | 1.74 | 442.7 | [C_30_H_50_O_2_](https://pubchem.ncbi.nlm.nih.gov/#query=C30H50O2) |
|  | Isoindolo(1,2-b)quinazolin-10(12H)-one | 28.458 | 0 | 0.76 | 234.25 | [C_15_H_10_N_2_O](https://pubchem.ncbi.nlm.nih.gov/#query=C15H10N2O) |
|  | Clionasterol acetate | 28.611 | 4651-54-1 | 1.47 | 456.7 | [C_31_H_52_O_2_](https://pubchem.ncbi.nlm.nih.gov/#query=C31H52O2) |
|  | 6-methoxy-2,7,8-trimethyl-2-(4,8,12 trimethyltridecyl) chroman | 28.737 | 79306-82-4 | 1.94 | 430.7 | [C_29_H_50_O_2_](https://pubchem.ncbi.nlm.nih.gov/#query=C29H50O2) |
|  | (2F,6E)-3,7,11-trimethyldodeca-2,6,10-trien-1-yl dodecanoate | 29.288 | 78368-58-8 | 0.66 | 404.67 | [C_27_H_48_O_2_](https://pubchem.ncbi.nlm.nih.gov/#query=C29H50O2) |
|  | 5-acetoxymethyl-2,6,10-trimethyl-2,9-undecadien-6-ol | 29.451 | 0 | 0.09 | 282.4 | [C_17_H_30_O_3_](https://pubchem.ncbi.nlm.nih.gov/#query=C17H30O3) |
|  | Ergost-5-en-3-ol, (3β)- | 30.063 | 4651-51-8 | 1.57 | 400.7 | [C_28_H_48_O](https://pubchem.ncbi.nlm.nih.gov/#query=C28H48O) |
|  | Cyclopropyl 2-(5’-methyl-2’-furyl) cyclopropyl ketone | 30.512 | 0 | 0.32 | 190.24 | [C_12_H_14_O_2_](https://pubchem.ncbi.nlm.nih.gov/#query=C12H14O2) |
