# Supplementary material for: Phytochemical screening and antibacterial activity of Skimmia anquetilia N.P. Taylor and Airy Shaw: A first study from Kashmir Himalaya
Source: Front Plant Sci. 2022 Aug 12;13:937946. doi: 10.3389/fpls.2022.937946 (PMC9412939; doi:10.3389/fpls.2022.937946)
Supplement: Supplementary file 3 [file Table_3.docx]

**Table S3. Bioactive compounds from the methanolic root extract of *Skimmia anquetilia***

| **S. No.** | **Compounds** | **Retention time (min)** | **CAS Number** | **Peak Area (%)** | **Molecular weight (g/mol)** | **Chemical formula** |
| --- | --- | --- | --- | --- | --- | --- |
|  | Glyceraldehydes | 4.388 | 56-82-6 | 0.24 | 90.08 | [C_3_H_6_O_3_](https://pubchem.ncbi.nlm.nih.gov/#query=C3H6O3) |
|  | 3-furaldehyde | 4.616 | 498-60-2 | 0.08 | 96.08 | [C_5_H_4_O_2_](https://pubchem.ncbi.nlm.nih.gov/#query=C5H4O2) |
|  | Dihydroxyacetone | 5.521 | 96-26-4 | 0.37 | 90.08 | [C_3_H_6_O_3_](https://pubchem.ncbi.nlm.nih.gov/#query=C3H6O3) |
|  | 2-hydroxy-2-cyclopenten-1-one | 6.262 | 10493-98-8 | 0.06 | 98.1 | [C_5_H_6_O_2_](https://pubchem.ncbi.nlm.nih.gov/#query=C5H6O2) |
|  | Glycerin | 7.092 | 56-81-5 | 0.10 | 92.09 | [C_3_H_8_O_3_](https://pubchem.ncbi.nlm.nih.gov/#query=C3H8O3) |
|  | Hexanoic acid | 7.262 | 142-62-1 | 0.09 | 116.15 | C_6_H_12_O_2_ |
|  | 2-hydroxy-γ-butyrolactone | 7.551 | 19444-84-9 | 0.08 | 102.09 | [C_4_H_6_O_3_](https://pubchem.ncbi.nlm.nih.gov/#query=C4H6O3) |
|  | Thymine | 9.357 | 65-71-4 | 0.06 | 126.11 | C_5_H_6_N_2_O_2_ |
|  | Linalool | 9.953 | 78-70-6 | 0.06 | 154.24 | C_10_H_18_O |
|  | 4H-pyran-4-one, 2,3-dihydro-3,5-dihydroxy-6-methyl- | 10.861 | 28564-83-2 | 0.11 | 144.12 | [C_6_H_8_O_4_](https://pubchem.ncbi.nlm.nih.gov/#query=C6H8O4) |
|  | 1-undecanol | 11.840 | 112-42-5 | 0.06 | 172.31 | [C_11_H_24_O](https://pubchem.ncbi.nlm.nih.gov/#query=C11H24O) |
|  | DL-2,3-butanediol | 12.201 | 6982-25-8 | 0.07 | 90.12 | [C_4_H_10_O_2_](https://pubchem.ncbi.nlm.nih.gov/#query=C4H10O2) |
|  | 5-hydroxymethylfurfural | 12.514 | 67-47-0 | 0.22 | 126.11 | [C_6_H_6_O_3_](https://pubchem.ncbi.nlm.nih.gov/#query=C6H6O3) |
|  | Geraniol | 13.112 | 106-24-1 | 0.08 | 154.25 | [C_10_H_18_O](https://pubchem.ncbi.nlm.nih.gov/#query=C10H18O) |
|  | Benzene, 1-(1-methylethenyl)-2-(1-methylethyl)- | 13.663 | 5557-93-7 | 0.06 | 160.25 | [C_12_H_16_](https://pubchem.ncbi.nlm.nih.gov/#query=C12H16) |
|  | 2-methoxy-4-vinylphenol | 14.340 | 7786-61-0 | 0.33 | 150.17 | [C_9_H_10_O_2_](https://pubchem.ncbi.nlm.nih.gov/#query=C9H10O2) |
|  | 2(3H)-furanone, 5-methyl- | 14.394 | 591-12-8 | 0.08 | 98.1 | [C_5_H_6_O_2_](https://pubchem.ncbi.nlm.nih.gov/#query=C5H6O2) |
|  | (Z)-3,7-dimethyl-2,6-octadienyl heptanoate | 15.221 | 0 | 0.09 | 266.4 | C_17_H_30_O_2_ |
|  | Geranyl acetate | 15.571 | 105-87-3 | 0.13 | 196.28 | C_12_H_20_O_2_ |
|  | Mandelic acid, 3,4-dimethoxy-, methyl ester | 15.989 | 2911-73-1 | 0.21 | 226.23 | [C_11_H_14_O_5_](https://pubchem.ncbi.nlm.nih.gov/#query=C11H14O5) |
|  | Cyclohexene, 1-(1-propynyl)- | 16.692 | 1655-05-6 | 0.52 | 120.19 | C_9_H_12_ |
|  | β-D-glucopyranose, 1,6-anhydro- | 17.268 | 498-07-7 | 0.10 | 162.14 | [C_6_H_10_O_5_](https://pubchem.ncbi.nlm.nih.gov/#query=C6H10O5) |
|  | 6-fluoro-2 trifluoromethylbenzoic acid, 2-formyl-4,6-dichlorophenyl ester | 17.911 | 0 | 0.45 | 381.106 | C_15_H_6_Cl_2_F_4_O_3_ |
|  | Dodecanoic acid, 2-methyl- | 18.078 | 2874-74-0 | 0.33 | 214.34 | [C_13_H_26_O_2_](https://pubchem.ncbi.nlm.nih.gov/#query=C13H26O2) |
|  | [Phenol, 2-(3-hydroxy-3-methyl-1-Butenyl)-, (Z)-](https://pubchem.ncbi.nlm.nih.gov/compound/5372791) | 18.122 | 17235-14-2 | 0.13 | 178.23 | [C_11_H_14_O_2_](https://pubchem.ncbi.nlm.nih.gov/#query=C11H14O2) |
|  | Nonanedioic acid, dimethyl ester | 18.476 | 1732-10-1 | 0.06 | 216.27 | [C_11_H_20_O_4_](https://pubchem.ncbi.nlm.nih.gov/#query=C11H20O4) |
|  | 1,2-pentadiene, 4-methoxy-4-methyl- | 18.673 | 49833-91-2 | 0.07 | 112.17 | [C_7_H_12_O](https://pubchem.ncbi.nlm.nih.gov/#query=C7H12O) |
|  | 1,3,3-trimethyl-2-hydroxymethyl-3,3-dimethyl-4-(3-methylbut-2-enyl)-cyclohexene | 18.809 | 0 | 0.41 | 222.37 | C_15_H_26_O |
|  | [Phenol, 4-ethenyl-2,6-dimethoxy](https://pubchem.ncbi.nlm.nih.gov/compound/35960) | 18.853 | 28343-22-8 | 0.82 | 180.2 | [C_10_H_12_O_3_](https://pubchem.ncbi.nlm.nih.gov/#query=C10H12O3) |
|  | [Benzenecarbothioic acid, 2,4,6-triethyl-, S-(2-phenylethyl) ester](https://pubchem.ncbi.nlm.nih.gov/compound/605137) | 19.129 | 64712-67-0 | 0.05 | 326.5 | [C_21_H_26_OS](https://pubchem.ncbi.nlm.nih.gov/#query=C21H26OS) |
|  | Heptane, 3,3-dimethyl- | 19.353 | 4032-86-4 | 0.06 | 128.25 | C_9_H_20_ |
|  | Pentanoic acid, 2-(aminooxy)- | 19.486 | 5699-55-8 | 0.21 | 133.15 | [C_5_H_11_NO_3_](https://pubchem.ncbi.nlm.nih.gov/#query=C5H11NO3) |
|  | 4,8-dioxatricyclo[5.1.0.0(3,5)octane,1-methyl-5-(1-methylethyl)-, (1a,3a,5a,7a)- | 19.986 | 42569-58-4 | 0.10 | 168.2 | [C_10_H_16_O_2_](https://pubchem.ncbi.nlm.nih.gov/#query=C10H16O2) |
|  | 3-phenyl-4-hydroxyacetophenone | 20..074 | 21424-82-8 | 0.59 | 212.24 | [C_14_H_12_O_2_](https://pubchem.ncbi.nlm.nih.gov/#query=C14H12O2) |
|  | Methyl *cis*-cinnamate | 20.993 | 19713-73-6 | 0.17 | 162.19 | C_10_H_10_O_2_ |
|  | 1,15-pentadecanediol | 21.207 | 14722-40-8 | 0.14 | 244.41 | C_15_H_32_0_2_ |
|  | Trans-farnesol | 21.353 | 106-28-5 | 0.65 | 222.37 | [C_15_H_26_O](https://pubchem.ncbi.nlm.nih.gov/#query=C15H26O) |
|  | (E)-4-(3-hydroxyprop-1-en-1-yl)-2-methoxyphenol | 21.632 | 32811-40-8 | 0.16 | 180.20 | [C_10_H_12_O_3_](https://www.chemeo.com/search?q=C10H12O3) |
|  | [2-naphthoic acid, 6-hydroxy-5,7-dimethoxy-, acetate](https://pubchem.ncbi.nlm.nih.gov/compound/619193) | 21.771 | 23673-54-3 | 0.09 | 290.27 | [C_15_H_14_O_6_](https://pubchem.ncbi.nlm.nih.gov/#query=C15H14O6) |
|  | Tricyclo[3.3.1.1(3,7)]decanone, 4-iodo-, (1a,3ß,4a,5a,7ß)- | 21.890 | 56781-85-2 | 0.09 | 276 | C_10_H_13_IO |
|  | 1,2-naphthalenedione, 6-hydroxy- | 21.945 | 607-20-5 | 0.07 | 174.15 | C_10_H_6_O_3_ |
|  | Photocitral B | 22.023 | 6040-45-5 | 0.08 | 152.23 | C_10_H_16_O |
|  | 2-quinolinecarboxylic acid, 4,6-dihydroxy- | 22.251 | 3778-29-8 | 0.17 | 205.17 | [C_10_H_7_NO_4_](https://pubchem.ncbi.nlm.nih.gov/#query=C10H7NO4) |
|  | 10-heneicosene (c,t) | 22.377 | 95008-11-0 | 0.16 | 294.56 | [C_21_H_42_](https://www.chemeo.com/search?q=C21H42) |
|  | 3-ethyl-2,6,10-trimethylundecane | 22.475 | 0 | 0.01 | 226.44 | [C_16_H_34_](https://pubchem.ncbi.nlm.nih.gov/#query=C16H34) |
|  | 9-dodecenoic acid, methyl ester, (E)- | 22.625 | 55030-26-7 | 0.10 | 212.33 | [C_13_H_24_O_2_](https://www.chemeo.com/search?q=C13H26O2) |
|  | Ambrial | 22.809 | 3243-36-5 | 0.19 | 234.37 | C_16_H_26_O |
|  | 3-cyanopyrazolo[3,4-d]pyrimidine-4-one | 22.935 | 5387-84-8 | 0.14 | 161.12 | [C_6_H_3_N_5_O](https://pubchem.ncbi.nlm.nih.gov/#query=C6H3N5O) |
|  | Farnesyl butanoate | 23.128 | 51532-27-5 | 1.41 | 292.46 | [C_19_H_32_O_2_](https://www.chemeo.com/search?q=C19H32O2) |
|  | 7-hydroxycoumarin | 23.220 | 93-35-6 | 0.49 | 162.14 | [C_9_H_6_O_3_](https://www.chemeo.com/search?q=C9H6O3) |
|  | Allyl hyponitrite | 23.438 | 118728-49-7 | 0.05 | 142.16 | [C_6_H_10_N_2_O_2_](https://pubchem.ncbi.nlm.nih.gov/#query=C6H10N2O2) |
|  | Phthalic acid, hept-4-yl isobutyl ester | 23.560 | 0 | 1.11 | 320.42 | C_19_H_28_O_4_ |
|  | Tetradecanoic acid, 10, 13-dimethyl-, methyl ester | 23.841 | 267650-23-7 | 1.31 | 270.45 | [C_17_H_34_O_2_](https://www.chemeo.com/search?q=C17H34O2) |
|  | [Bicyclo[5.2.1]decan-10-one](https://pubchem.ncbi.nlm.nih.gov/compound/566719) | 23.958 | 4696-15-5 | 0.09 | 152.23 | [C_10_H_16_O](https://pubchem.ncbi.nlm.nih.gov/#query=C10H16O) |
|  | 1-hexyl-2-nitrocyclohexane | 24.089 | 118252-04-3 | 15.43 | 213.32 | C_12_H_23_NO_2_ |
|  | Dibutyl phthalate | 24.227 | 84-74-2 | 0.11 | 278.34 | [C_16_H_22_O_4_](https://pubchem.ncbi.nlm.nih.gov/#query=C16H22O4) |
|  | Hexadecanoic acid, methyl ester | 24.309 | 112-39-0 | 7.24 | 270.45 | C_17_H_34_O_2_ |
|  | 2H-inden-2-one, 1,4,5,7a-tetrahydro-6,7-bis(hydroxymethyl)- | 24.513 | 55759-92-7 | 0.28 | 194.23 | C_11_H_14_O_3_ |
|  | 2-naphthoic acid, 6-hydroxy-5,7-dimethoxy-, acetate | 24.703 | 23673-54-3 | 0.66 | 290.27 | C_15_H_14_O_6_ |
|  | *n*-hexadecanoic acid | 24.809 | 57-10-3 | 1.35 | 256.42 | [C_16_H_32_O_2_](https://pubchem.ncbi.nlm.nih.gov/#query=C16H32O2) |
|  | 4-acetylbenzoic acid | 24.945 | 586-89-0 | 0.93 | 164.16 | C_9_H_8_O_3_ |
|  | 4-nonene, 3-methyl-, (Z)- | 25.009 | 63830-69-3 | 0.34 | 140.27 | C_10_H_20_ |
|  | 2H-1-benzopyran-2-one, 7-hydroxy-6-methoxy-4-methyl- | 25.091 | 3374-03-6 | 0.21 | 206.19 | [C_11_H_10_O_4_](https://pubchem.ncbi.nlm.nih.gov/#query=C11H10O4) |
|  | 1-dodecanol, 3,7,11-trimethyl- | 25.241 | 6750-34-1 | 0.42 | 228.41 | C_15_H_32_O |
|  | [Tetradecanoic acid, 12 methyl-, methyl ester](https://pubchem.ncbi.nlm.nih.gov/compound/23618376), (S)- | 25.305 | 62691-05-8 | 4.54 | 256.42 | [C_16_H_32_O_2_](https://pubchem.ncbi.nlm.nih.gov/#query=C16H32O2) |
|  | (E)-15,16-dinorlabda-8(17), 11-dien-13-one | 25.421 | 76497-69-3 | 0.28 | 260.41 | [C_18_H_28_O](https://www.chemeo.com/search?q=C18H28O) |
|  | [9,11-octadecadiynoic acid, 8-oxo-, methyl ester](https://pubchem.ncbi.nlm.nih.gov/compound/595093) | 25.485 | 75125-35-8 | 0.36 | 304.4 | [C_19_H_28_O_3_](https://pubchem.ncbi.nlm.nih.gov/#query=C19H28O3) |
|  | 1-methylene-2b-hydroxymethyl-3,3-dimethyl-4b-(3methylbut-2-enyl)-cyclohexane | 25.771 | 0 | 1.78 | 222.37 | [C_15_H_26_O](https://pubchem.ncbi.nlm.nih.gov/#query=C15H26O) |
|  | [(2S,4S)-2,4-dimethylheptanedioic acid dimethyl ester](https://pubchem.ncbi.nlm.nih.gov/compound/22211749) | 26.210 | 85611-45-6 | 0.32 | 216.27 | [C_11_H_20_O_4_](https://pubchem.ncbi.nlm.nih.gov/#query=C11H20O4) |
|  | 7H-furo[3,2-g][1] benzopyran-7-one, 4-methoxy- | 26.353 | 484-20-8 | 1.28 | 216.19 | [C_12_H_8_O_4_](https://pubchem.ncbi.nlm.nih.gov/#query=C12H8O4) |
|  | Methyl 8- methoxy-1-methyl-2,3,4,9- tetrahydro-1H-beta-  carboline-1-carboxylate# | 26.444 | 112513-54-9 | 0.05 | 274.31 | C_15_H_18_N_2_O_3_ |
|  | [Methyl-methoxy-hydroxymethyl-amine](https://pubchem.ncbi.nlm.nih.gov/compound/554060) | 26.530 | 6919-52-4 | 0.07 | 91.11 | [C_3_H_9_NO_2_](https://pubchem.ncbi.nlm.nih.gov/#query=C3H9NO2) |
|  | Methyl 9-*cis*, 11-trans-octadecadienoate | 26.621 | 13058-52-1 | 9.62 | 294.47 | C_19_H_34_O_2_ |
|  | *cis*,*cis*,*cis*-7,10,13-hexadecatrienal | 26.706 | 56797-43-4 | 13.29 | 234.39 | C_16_H_26_O |
|  | Undecane, 2,6-dimethyl- | 26.938 | 17301-23-4 | 0.07 | 184.36 | [C_13_H_28_](https://www.chemeo.com/search?q=C13H28) |
|  | 5, 10-pentadecadien-1-ol, (Z,Z)- | 27.152 | 64275-51-0 | 6.28 | 224.38 | [C_15_H_28_O](https://pubchem.ncbi.nlm.nih.gov/#query=C15H28O) |
|  | 9(E), 11(E)-conjugated linoleic acid, ethyl ester | 27.478 | 0 | 1.28 | 280.5 | C_18_H_32_O_2_ |
|  | [2,2,6-trimethyl-2H,5H-pyrano[3,2-c]quinolin-5-one](https://pubchem.ncbi.nlm.nih.gov/compound/72819) | 27.665 | 50333-13-6 | 0.23 | 241.28 | [C_15_H_15_NO_2_](https://pubchem.ncbi.nlm.nih.gov/#query=C15H15NO2) |
|  | 5, 10-pentadecadiyne, 1-chloro- | 27.944 | 64275-44-1 | 0.66 | 238.79 | [C_15_H_23_Cl](https://pubchem.ncbi.nlm.nih.gov/#query=C15H23Cl) |
|  | 2H-furo[2,3-h]-1-benzopyran-2-one-, 8-(1-methylethenyl)- | 28.230 | 1760-27-6 | 0.24 | 226.23 | [C_14_H_10_O_3_](https://pubchem.ncbi.nlm.nih.gov/#query=C14H10O3) |
|  | Benzoic acid, 4-(4-propylcyclohexyl)-, 4-cyano-4-ethylcyclohexyl ester | 28.295 | 0 | 0.34 | 423.5 | [C_29_H_29_NO_2_](https://pubchem.ncbi.nlm.nih.gov/#query=C29H29NO2) |
|  | 7H-furo[3,2-g][1]benzopyran-7-one,4,9-dimethoxy- | 28.618 | 482-27-9 | 2.62 | 246.21 | [C_13_H_10_O_5_](https://pubchem.ncbi.nlm.nih.gov/#query=C13H10O5) |
|  | 3,4-nonadien-6-yne,5-ethyl-3-methyl- | 28.795 | 61227-88-1 | 0.39 | 162.28 | C_12_H_18_ |
|  | [3-picoline, 6-(tert-butylthio)](https://pubchem.ncbi.nlm.nih.gov/compound/580513) | 28.880 | 18794-46-2 | 0.13 | 181.3 | [C_10_H_15_NS](https://pubchem.ncbi.nlm.nih.gov/#query=C10H15NS) |
|  | 2,3,4-trimethoxydibenzofuran | 28.999 | 88256-11-5 | 0.46 | 258.26 | [C_15_H_14_O_4_](https://pubchem.ncbi.nlm.nih.gov/#query=C15H14O4) |
|  | 1-methylene-2b-hydroxymethyl-3,3-dimethyl-4b-(3-methylbut-2-enyl)-cyclohexane | 29.159 | 0 | 1.10 | 222.37 | C_15_H_26_O |
|  | 1-hexyl-1-nitrocyclohexane | 29.261 | 118252-09-8 | 0.86 | 213.32 | C_12_H_23_NO_2_ |
|  | 5, 10-pentadecandiyn-1-ol | 29.339 | 64275-50-9 | 0.33 | 220.35 | [C_15_H_24_O](https://pubchem.ncbi.nlm.nih.gov/#query=C15H24O) |
|  | 9,11-octadecadiynoic acid, 8-oxo-, methyl ester | 29.696 | 75125-35-8 | 0.45 | 304.4 | [C_19_H_28_O_3_](https://pubchem.ncbi.nlm.nih.gov/#query=C19H28O3) |
|  | [Cyclohexanol, 1-ethyl-2,2-dimethyl-6-methylene-](https://pubchem.ncbi.nlm.nih.gov/compound/567152) | 29.863 | 54345-64-1 | 0.11 | 168.28 | [C_11_H_20_O](https://pubchem.ncbi.nlm.nih.gov/#query=C11H20O) |
|  | 7H-furo[3,2-g][1]benzopyran-7-one, 9-[(3-methyl-2-butenyl)oxy]- | 29.971 | 482-44-0 | 0.50 | 270.28 | C_16_H_14_O_4_ |
|  | (E,E,E)-(5-phenylsulfonylgeranyl)geraniol | 30.104 | 67428-43-7 | 0.23 | 430.64 | C_26_H_38_O_3_S |
|  | Nonacos-1-ene | 30.244 | 18835-35-3 | 0.48 | 400.72 | C_29_H_52_ |
|  | Eicosanal- | 30.662 | 2400-66-0 | 0.23 | 296.5 | [C_20_H_40_O](https://pubchem.ncbi.nlm.nih.gov/#query=C20H40O) |
|  | 1-methyl-4-isopropyl-cyclohexyl 2-hydroperfluorobutanoate | 30.846 | 0 | 0.25 | 334.3 | [C_14_H_20_F_6_O_2_](https://pubchem.ncbi.nlm.nih.gov/#query=C14H20F6O2) |
|  | 4-(3-methyl-2-oxobutoxy)-7H-furo[3,2-g][1]benzopyran-7-one | 31.471 | 5058-15-1 | 0.12 | 286.27 | C_16_H_14_O_5_ |
|  | Hexadecanoic acid, 2-hydroxy-1-(hydroxymethyl)ethyl ester | 31.577 | 23470-00-0 | 1.50 | 330.50 | C_19_H_38_O_4_ |
|  | Glycerol 1-palmitate | 31.699 | 542-44-9 | 0.13 | 330.50 | C_19_H_38_O_4_ |
|  | Pthalic acid, di(2-propylpentyl)ester | 32.050 | 0 | 1.71 | 390.55 | C_24_H_38_O_4_ |
|  | Pentacos-1-ene | 32.182 | 16980-85-1 | 0.12 | 350.66 | C_25_H_50_ |
|  | 2-isopropyl-5-methylcyclohexyl 3-(1-(4-chlorophenyl)-3-oxobutyl)-coumarin-4-yl carbonate | 32.274 | 0 | 0.20 | 525 | [C_30_H_33_ClO_6_](https://pubchem.ncbi.nlm.nih.gov/#query=C30H33ClO6) |
|  | 1-allyl-cyclohexane-1,2-diol | 32.413 | 0 | 0.53 | 156.22 | [C_9_H_16_O_2_](https://pubchem.ncbi.nlm.nih.gov/#query=C9H16O2) |
|  | Formaldehyde, methyl(2-propenyl)hydrazone | 32.703 | 66075-09-0 | 0.10 | 98.15 | [C_5_H_10_N_2_](https://pubchem.ncbi.nlm.nih.gov/#query=C5H10N2) |
|  | 2H-1-benzopyran-2-one, 7-[(3,7-dimethyl-2,6-octadienyl)oxy]-, (E)- | 32.975 | 495-02-3 | 1.30 | 298.4 | C_19_H_22_O_3_ |
|  | [4-[(2-thiazolyl)azo]orcinol](https://pubchem.ncbi.nlm.nih.gov/compound/616616) | 33.396 | 37422-56-3 | 0.16 | 235.26 | [C_10_H_9_N_3_O_2_S](https://pubchem.ncbi.nlm.nih.gov/#query=C10H9N3O2S) |
|  | Wampetin | 33.713 | 89824-26-0 | 0.60 | 366.4 | [C_21_H_18_O_6_](https://pubchem.ncbi.nlm.nih.gov/#query=C21H18O6) |
|  | [2,3-heptadien-5-yne, 2,4-dimethyl-](https://pubchem.ncbi.nlm.nih.gov/compound/570698) | 34.352 | 41898-89-9 | 0.11 | 120.19 | [C_9_H_12_](https://pubchem.ncbi.nlm.nih.gov/#query=C9H12) |
|  | [4,5,9-trihydroxy-dodeca-1,11-diene](https://pubchem.ncbi.nlm.nih.gov/compound/534592) | 34.699 | 121231-46-7 | 0.06 | 214.3 | [C_12_H_22_O_3_](https://pubchem.ncbi.nlm.nih.gov/#query=C12H22O3) |
|  | Squalene | 35.053 | 111-02-4 | 0.56 | 410.72 | C_30_H_50_ |
|  | Acetanide, N-(4-methylphenyl)-N-[4-methyl-2-[[2-  (phenylamino)phenyl]methyl]phenyl]- | 35.454 | 52812-78-9 | 0.09 | 420.5 | C_29_H_28_N_2_O |
|  | Ethanone, 1,1’-(6-methoxy-2,5-benzofurandiyl)bis- | 36.042 | 23840-15-5 | 0.13 | 232.23 | [C_13_H_12_O_4_](https://pubchem.ncbi.nlm.nih.gov/#query=C13H12O4) |
|  | [Ergost-5-en-3-ol, acetate, (3β,24R)-](https://pubchem.ncbi.nlm.nih.gov/compound/13019955) | 37.804 | 0 | 0.13 | 442.7 | [C_30_H_50_O_2_](https://pubchem.ncbi.nlm.nih.gov/#query=C30H50O2) |
